# Modularity and three-dimensional isostructurality of novel synthons in sulfonamide–lactam cocrystals

**DOI:** 10.1107/S2052252515004960

**Published:** 2015-05-01

**Authors:** Geetha Bolla, Sudhir Mittapalli, Ashwini Nangia

**Affiliations:** a School of Chemistry, University of Hyderabad, Prof. C. R. Rao Road, Central University PO, Hyderabad, 500 046, India

**Keywords:** crystal engineering, supramolecular synthons, pharmaceutical cocrystals, isostructurality

## Abstract

The design of novel supramolecular synthons for functional groups relevant to drugs is an essential prerequisite for applying crystal engineering in the development of novel pharmaceutical cocrystals. It has been convincingly shown over the past decade that molecular level control and modulation can influence the physicochemical properties of drug cocrystals. Whereas considerable advances have been reported on the design of cocrystals for carboxylic acids and carboxamide functional groups, the sulfonamide group, which is a cornerstone of sulfa drugs, is relatively unexplored for reproducible heterosynthon-directed crystal engineering. The occurrence of synthons and isostructurality in sulfonamide–lactam cocrystals (SO_2_NH_2_⋯CONH hydrogen bonding) is analyzed to define a strategy for amide-type GRAS (generally recognized as safe) coformers with sulfonamides. Three types of supramolecular synthons are identified for the N—H donor of sulfonamide hydrogen bonding to the C=O acceptor of amide. Synthon 1: catemer synthon *C*_2_^1^(4) chain motif, synthon 2: dimer–cyclic ring synthon *R*_2_^2^(8)*R*_4_^2^(8) motifs, and synthon 3: dimer–catemer synthon of *R*_2_^2^(8)*C*_1_^1^(4)*D* notation. These heterosynthons of the cocrystals observed in this study are compared with the N—H⋯O dimer *R*_2_^2^(8) ring and *C*(4) chain motifs of the individual sulfonamide structures. The X-ray crystal structures of sulfonamide–lactam cocrystals exhibit interesting isostructurality trends with the same synthon being present. One-dimensional, two-dimensional and three-dimensional isostructurality in crystal structures is associated with isosynthons and due to their recurrence, novel heterosynthons for sulfonamide cocrystals are added to the crystal engineer’s toolkit. With the predominance of sulfa drugs in medicine, these new synthons provide rational strategies for the design of binary and potentially ternary cocrystals of sulfonamides.

## Abstract 2

Novel supramolecular synthons for primary sulfonamides with cyclic amides (GRAS coformers) are introduced for the crystal engineering of pharmaceutical cocrystals of sulfa drugs. The cocrystals of this model study exhibit isostructurality and isosynthons mediated by SO_2_NH_2_⋯CONH hydrogen bonding.

## 1. Introduction   

The concept of supramolecular synthons introduced by Desiraju in 1995 (Desiraju, 1995 ▸; Thalladi *et al.*, 1996 ▸; Reddy *et al.*, 1996 ▸; Dunitz & Gavezzotti, 2012 ▸; Nangia & Desiraju, 1998 ▸) led to the identification of known and new hydrogen bond patterns in crystal engineering. Zaworotko and coworkers (Walsh *et al.*, 2003 ▸) sub-classified synthons as homosynthons (those between like functional groups) and heterosynthons (hydrogen bonds between unlike groups). The past decade has witnessed immense interest in utilizing various supramolecular synthons to direct structural organization in the crystal structure. For example, acid–acid and amide–amide homosynthons are well known, while acid–pyridine and acid–amide are popular heterosynthons. The latter form of association between unlike functional groups has immediate potential in the engineering of multi-component systems, notably cocrystals (Vishweshwar *et al.*, 2003*a* ▸,*b* ▸; Biradha & Zaworotko, 1998 ▸; Bis & Zaworotko, 2005 ▸; Bis *et al.*, 2006 ▸; Vangala *et al.*, 2005 ▸; Ermer & Eling, 1994 ▸; Reddy *et al.*, 2006 ▸, 2007 ▸; Babu *et al.*, 2007 ▸; Goud *et al.*, 2011 ▸; Kaur & Guru Row, 2012 ▸)

**Figure 1:**
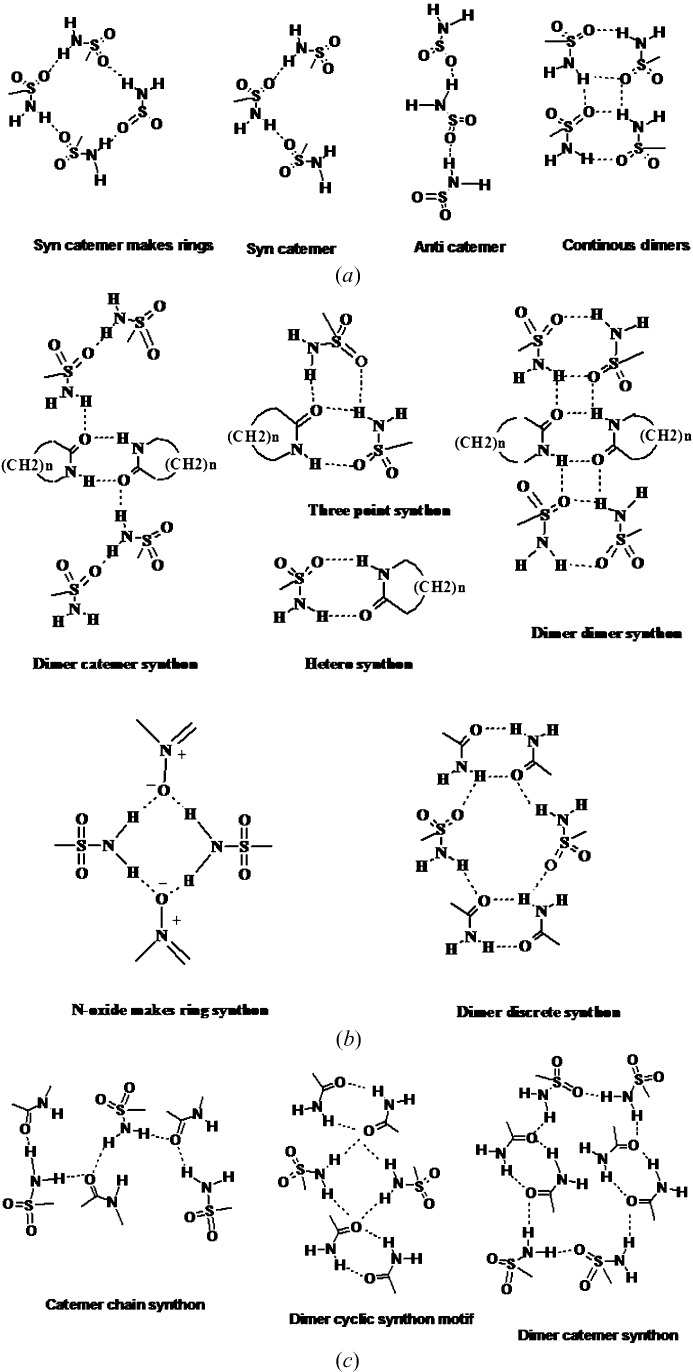
(*a*) Synthons present in primary sulfonamides (homosynthons). (*b*) Synthon motifs present in sulfonamide cocrystals (heterosynthons) from the literature study. (*c*) Synthon motifs present in sulfonamide cocrystals discussed in this report (heterosynthons).

Selected homo- and heterosynthons extracted from the literature for single and multi-component systems of sulfonamides are listed in Fig. 1 ▸. The directionality and strength of hydrogen bonding plays a major role in controlling the supramolecular assembly through complementary functional groups, which leads to the application of crystal engineering in material science and pharmaceutical solids (Childs *et al.*, 2004 ▸; Trask, Motherwell & Jones, 2004 ▸, 2005 ▸, 2006 ▸; Trask, Haynes *et al.*, 2006 ▸). The pairing of best-donor to best-acceptor hydrogen bonding (Etter’s rules) guides cocrystal design in a majority of cases (Etter, 1982 ▸, 1990 ▸, 1991 ▸). However, as multiple functional groups come into interplay, the competition can be more complex and difficult to predict (Sarma *et al.*, 2009 ▸; Aakeröy *et al.*, 2013 ▸). For this reason, we examined heterosynthons of sulfonamides with the amide group in non-competing binary systems with the idea of developing a library of sulfonamide–carboxamide synthons. Whereas sulfonamide and carboxamide homosynthons have been studied, this is a report on their heterosynthons. Sulfon­amides preferably form dimer and catemer synthons, whereas carboxamides more often assemble *via* the dimer synthon (Sanphui *et al.*, 2010 ▸). A robust heterosynthon for sulfonamide group cocrystals is that with pyridine *N*-oxides (Goud *et al.*, 2011 ▸), but since the latter coformers are not pharmaceutically acceptable, there is a need to develop a design strategy for sulfonamides with GRAS amides (US-FDA, 2014 ▸). Selected data on sulfonamides were extracted from the Cambridge Structural Database (CSD, Version 5.36, November 2014 release). With this background, benzene sulfonamides were cocrystallized with cyclic carboxamides to analyze isostructural relationships and classify the observed synthons.

**Figure 2:**
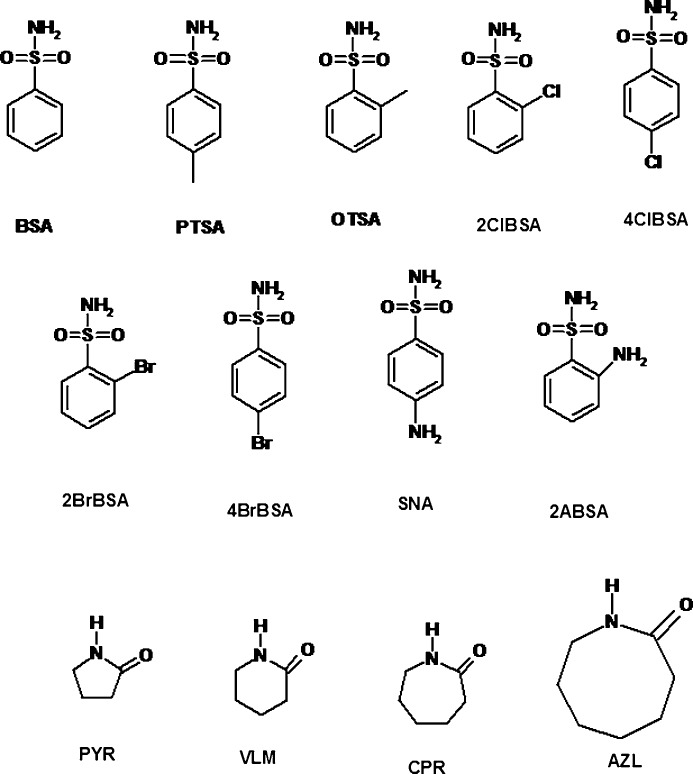
Molecular structure of the primary sulfonamides and lactams used in this study to make binary cocrystals.

Primary sulfonamides attached to a substituted phenyl ring were selected in this exploratory cocrystal study to identify the basic heterosynthons with amides in a non-competitive environment. We were successful in obtaining cocrystals of a few benzene sulfonamides with lactams (*syn* amides) listed in Fig. 2 ▸. A reason to choose cyclic amides over primary amides was that the latter have *syn* and *anti* N—H donors, and together with primary sulfonamide, which also has *syn* and *anti* N—H donors, the diversity of hydrogen bond motifs may become too complex for systematic analysis. In a recent study of lactams with carboxylic acids, Moragues-Bartolome *et al.* (2012 ▸) found that 2-pyrrolidone showed a heterotetramer (CONH⋯COOH), whereas δ-valerolactam has a homotetramer synthon (CONH⋯CONH), although there were some mixed results as well (Moragues-Bartolome *et al.*, 2012 ▸). We report in this paper isostructural pairs of cocrystals (sulfonamide–lactam) having isosynthons (similar supramolecular synthons). The lattice parameters and crystal packing of the X-ray crystal structures suggest that there are three sets of isostructural compounds and that each set has its own isosynthons. Primary sulfonamides consist of two acceptor O atoms and two donor H atoms (SO_2_NH_2_), and the complementary functional group lactam (HN—C=O) also has one donor and one acceptor.

## 2. Experimental   

### 2.1. Preparation of cocrystals   

All the benzene sulfonamides and coformers (caprolactam, valerolactam *etc.*) used in this study (see Fig. 2 ▸) were purchased from Sigma–Aldrich, Hyderabad, India, and used as such without further purification. Equivalent amounts of the sulfonamide and coformer were taken in a mortar and ground with a pestle for 20–30 min using solvent-assisted grinding by adding a few drops of EtOAc. After confirming that the ground mixture is a new solid phase by powder X-ray diffraction (PXRD), the mixture was dissolved in EtOAc or EtOAc–THF. The solution was then allowed to cocrystallize at room temperature by slow evaporation. Suitable crystals for single-crystal X-ray data were obtained after 5–6 d. A summary of the grinding experiments, characterizations of cocrystals by PXRD and IR, and confirmation by single-crystal X-ray diffraction (SC-XRD) are listed in Table 1 ▸.

### 2.2. BSA–VLM cocrystal (1:1)   

BSA (100 mg, 0.636 mmol) and VLM (63 mg, 0.636 mmol) were ground well in a mortar and pestle for 20–30 min by adding 4–7 drops of EtOAc (liquid-assisted grinding or LAG; Shan *et al.*, 2002 ▸; Trask & Jones, 2005 ▸; Friščić *et al.*, 2006 ▸). The ground material was kept for crystallization in 5 ml of an EtOAc–THF mixture as well as in individual solvents at room temperature. Good diffraction-quality crystals were harvested under ambient conditions after 3–4 d; m.p. 79–81°C.

### 2.3. BSA–CPR cocrystal (1:1)   

BSA (100 mg, 0.636 mmol) and CPR (72 mg, 0.636 mmol) were ground well in a mortar and pestle for 20–30 min by adding 4–7 drops of EtOAc. The ground material was kept for crystallization in 5 mL of an EtOAc–THF solvent mixture as well as in individual solvents in a 25 ml conical flask at room temperature. Good quality crystals were harvested under ambient conditions after 3–4 days; m.p. 80–83°C.

### 2.4. BSA–AZL (1-aza-2-cyclooctanone) cocrystal (1:1)   

BSA (100 mg, 0.636 mmol) and AZL (80.87 mg, 0.636 mmol) were ground well in a mortar and pestle for 20–30 min by adding 4–7 drops of EtOAc. The ground material was kept for crystallization in 5 mL of an EtOAc–THF mixture as well as individual solvents in a 25 ml conical flask at room temperature. Good quality crystals were harvested under ambient conditions after 3–4 days; m.p. 76–81°C.

### 2.5. 2ClBSA–VLM, 4ClBSA–VLM cocrystal (1:1)   

ClBSA isomer (100 mg, 0.521 mmol) and VLM (51.6 mg, 0.521 mmol) were ground well in a mortar and pestle for 20–30 min by adding 5 drops of EtOAc. The ground material was kept for crystallization in 5 mL of an EtOAc–THF mixture as well as individual solvents at room temperature. Single crystals were harvested under ambient conditions after 3–4 d; m.p. 80–82°C; 90–91°C

### 2.6. 2ClBSA–CPR, 4ClBSA–CPR cocrystal (1:1)   

ClBSA isomer (100 mg, 0.521 mmol) and CPR (59 mg, 0.521 mmol) were ground well in a mortar and pestle for 20–30 min by adding 5 drops of EtOAc. The ground material was kept for crystallization in 5 mL of an EtOAc–THF mixture, as well as separate solvents at room temperature. Single crystals were harvested under ambient conditions after 3–4 d; m.p. 80–82°C; 82–83°C.

### 2.7. 4BrBSA–VLM cocrystal (1:1)   

4BrBSA (100 mg, 0.423 mmol) and VLM (51.6 mg, 0.423 mmol) were ground well in a mortar and pestle for 20–30 min with solvent assistance by adding 4–7 drops of EtOAc. The ground material was kept for crystallization in 5 mL of an EtOAc–THF mixture, as well as individual solvents at room temperature. Single crystals were harvested under ambient conditions after 3–4 d; m.p. 92–94°C.

### 2.8. 4BrBSA–CPR cocrystal (1:1)   

4BrBSA (100 mg, 0.423 mmol) and CPR (58.95 mg, 0.423 mmol) were ground well in a mortar aand pestle for 20–30 min through solvent-assisted grinding by adding 5 drops of EtOAc. The ground material was kept for crystallization in 5 mL of EtOAc–THF mixture as well as separate solvents. Single crystals were harvested at ambient conditions after 3–4 days; m.p. 90–92°C.

### 2.9. OTSA–VLM, PTSA–VLM cocrystal (1:1)   

OTSA/PTSA (100 mg, 0.584 mmol) and VLM (57.89 mg, 0.584 mmol) were ground well in a mortar and pestle for 20–30 min through solvent-assisted grinding by adding 5 drops of EtOAc. The ground material was kept for crystallization in 5 mL of an EtOAc–THF mixture as well as separate solvents. Single crystals were harvested under ambient conditions after 3–4 days; m.p. 70–72°C; 74–75°C.

### 2.10. SNA–VLM, 2ABSA–VLM cocrystal (1:1)   

SNA/2ABSA (100 mg, 0.580 mmol) and VLM (65.63 mg, 0.580 mmol) were ground well in a mortar and pestle for 20–30 min through solvent-assisted grinding by adding 5 drops of EtOAc. The ground material was kept for crystallization in 5 mL of an EtOAc–THF mixture as well as separate solvents. Single crystals were harvested at ambient conditions after 3–4 d; m.p. 95–97°C, 87–91°C.

### 2.11. Single-crystal X-ray diffraction   

A single crystal obtained from the crystallization experiment was mounted on the goniometer of an Oxford Diffraction Gemini X-ray diffractometer equipped with an Mo *K*α radiation source (λ = 0.71073 Å). Data reduction was performed using *CrysAlisPro* 171.33.55 software. The crystal structure was solved and refined using Olex2-1.0 with anisotropic displacement parameters for non-H atoms. H atoms were experimentally located through the difference-Fourier electron density maps in all crystal structures. Data was reduced by *SAINT-Plus* (Bruker, 1998 ▸) and further continued with *SHELXTL* (Sheldrick, 2008 ▸). A check of the final crystallographic information file (CIF) with *PLATON* (Spek, 2009 ▸) did not show any missed symmetry. *X-Seed* was used to prepare the figures and packing diagrams. Crystallographic parameters of all the cocrystals are summarized in Table 2 ▸. Hydrogen bond distances (see Table S1 of the supporting information) are neutron-normalized (O—H 0.983, N—H 0.82, C—H 1.083 Å). CIF files are also deposited with the CCDC (Nos. 1039188–1039200).

Some single-crystal diffraction data were collected at 298 K on a Bruker SMART APEX-1 CCD area-detector system equipped with a graphite monochromator, Mo *K*α fine-focus sealed tube (λ = 0.71073 Å) operated at 1500 W power (40 kV, 30 mA). The frames were integrated with *SAINT* (Bruker, 1998 ▸) software using a narrow-frame integration algorithm. Data was corrected for absorption effects using the multi-scan method (*SADABS*; Bruker, 1998 ▸). The structure was solved and refined using *SHELXTL* (Sheldrick, 2008 ▸).

### 2.12. X-ray powder diffraction   

Bulk samples were analyzed by X-ray powder diffraction on a Bruker AXS D8 diffractometer (Bruker-AXS, Karlsruhe, Germany). Experimental conditions: Cu *K*α radiation (λ = 1.54056 Å); 40 kV; 30 mA; scanning interval 5–50° 2θ at a scan rate of 1° min^−1^; time per step 0.5 s. The experimental PXRD patterns of the BSA, 4Cl BSA and 4Br BSA cocrystals were compared to confirm the isostructurality (Fig. S4 of the supporting information).

### 2.13. Vibrational spectroscopy   

A Thermo-Nicolet 6700 FT–IR spectrometer (Waltham, MA, USA) was used to record the IR spectra. IR spectra were recorded on samples dispersed in KBr pellets. For details of IR spectra see Fig. S8 and Table S4.

## 3. Results and discussion   

### 3.1. Crystal structure analysis and isostructurality   

**Figure 3:**
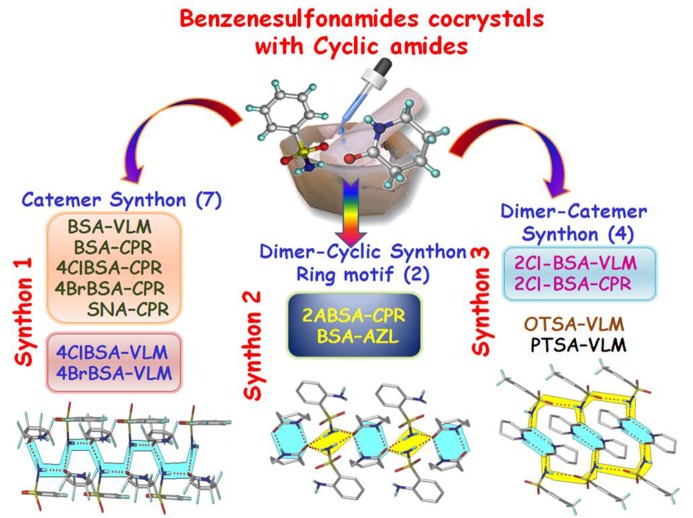
Classification of three novel synthons in sulfonamide–lactam cocrystals. Names of the cocrystal structures are shown in the bottom row.

A few benzene sulfonamides (listed in Fig. 2 ▸) were selected to make cocrystals with PYR, VLM, CPR and AZL cyclic amides in a 1:1 stoichiometric ratio, which were ground mechanochemically through solvent-assisted grinding to obtain cocrystals. The resulting binary systems were analyzed with greater emphasis on VLM and CPR cocrystals since they are pharmaceutically acceptable coformers. Three types of synthons were observed: synthon 1 or the catemer motif of graph-set *C*_2_^1^(4) (Etter *et al.*, 1990 ▸; Bernstein *et al.*, 1995 ▸), synthon 2 which is a dimer–cyclic synthon motif of *R*_2_^2^(8)*R*_4_^2^(8), and synthon 3 as a dimer–catemer motif *R*_2_^2^(8)*C*_1_^1^(4)*D* (Fig. 3 ▸). The crystal structure of BSA with AZL contains synthon 2. The crystal structures of other primary sulfonamides with AZL, PYR *etc.* will be discussed separately. Cocrystals of celecoxib (SO_2_NH_2_ drug) with odd/even homolog cyclic amides (Bolla *et al.*, 2014 ▸) indicated that the odd ring size coformer (PYR, CPR) follows the heterosynthon, whereas even ring lactams (VLM, AZL) result in dimer–dimer/dimer–catemer synthons. With the aim of establishing a trend for sulfonamides, this study however did not give the previously observed synthons but resulted in different motifs. A robust and predictable functional group for sulfonamide cocrystals is pyridine *N*-oxide coformers (as well as P- and As-oxide) (*e.g.* Goud *et al.*, 2011 ▸; Croker *et al.*, 2012 ▸; Ferguson *et al.*, 1989 ▸; Denise *et al.*, 2014 ▸), but these are not of practical use as pharmaceuticals since they are not GRAS molecules (generally regarded as safe). The cocrystals obtained in this study and synthon classification are summarized in Fig. 3 ▸, along with crystallographic parameters in Table 2 ▸ (additional data in Table 3 ▸).

### 3.2. Synthon 1, catemer chain   

Among the 13 cocrystal structures studied (Table 1 ▸), seven structures contain the sulfonamide–*syn*-carboxamide catemer synthon of *C*_2_^1^(4) notation. The catemer chains are assembled by sulfonamide N—H donors hydrogen bonding to the carboxamide acceptor. The structures are isostructural upon altering the auxiliary functional groups of benzene sulfonamide, such as Cl/Br/NH_2_/CH_3_. BSA–VLM and BSA–CPR have the same unit-cell parameters, whereas *p*-substituted BSA molecules (such as 4ClBSA, 4BrBSA and SNA) showed a 0.5 Å increase in the crystallographic *b*- and *c*-axis. BSA–VLM, BSA–CPR, SNA–CPR, 4ClBSA–CPR and 4BrBSA–CPR are three-dimensional isostructural. There are two more sets of isostructural cocrystals, 4ClBSA–VLM and 4BrBSA–VLM, with the same synthon.

#### 3.2.1. BSA–VLM, BSA–CPR, SNA–CPR, 4 ClBSA–CPR and 4BrBSA–CPR (1:1)   

**Figure 4:**
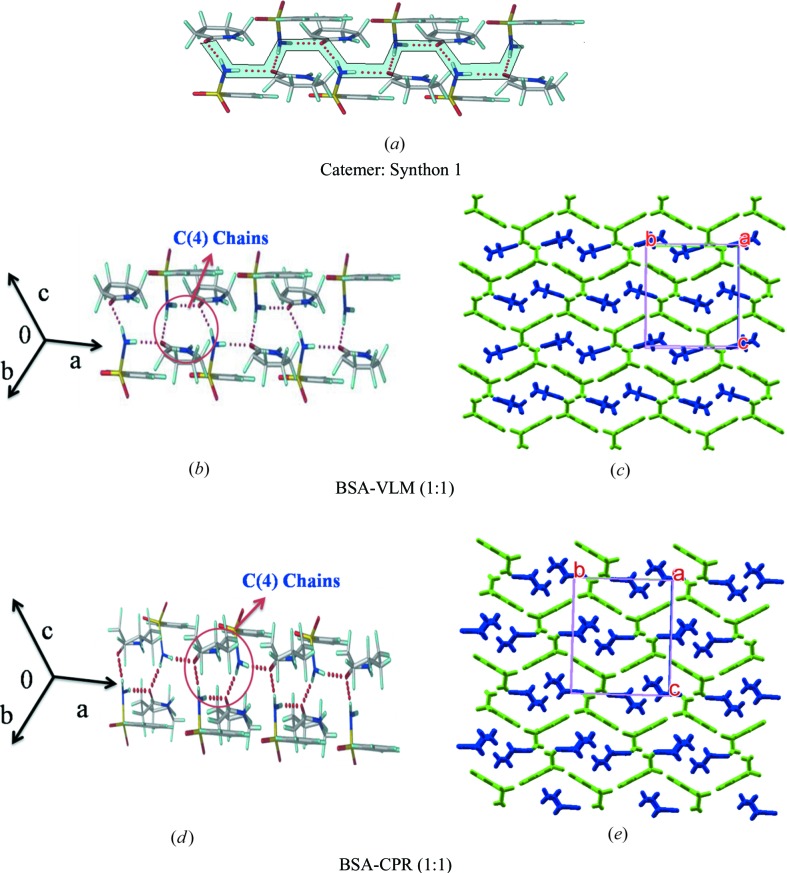

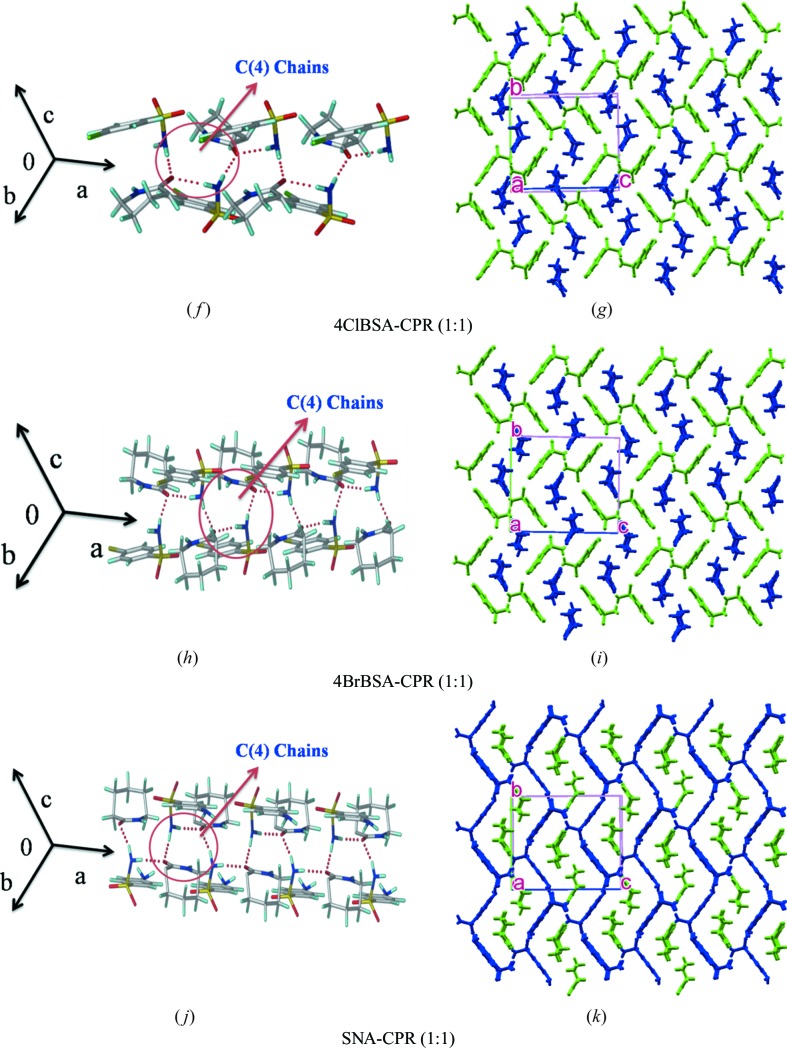

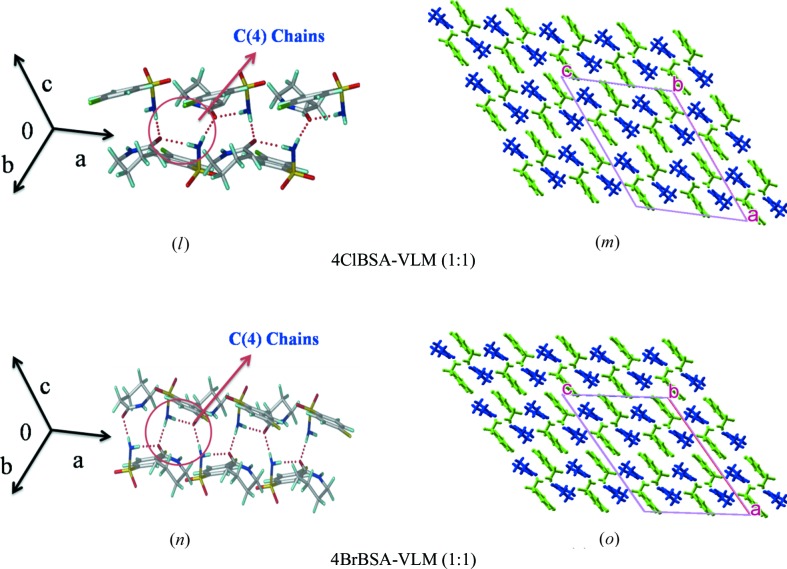
Crystal structures of sulfonamide–lactam cocrystals with catemer synthon 1. Two-dimensional packing diagrams are drawn with the asymmetric unit showing benzene sulfonamides (in green) and lactams (in blue) (VLM, CPR).

The crystal structures of all these multi-component systems were refined in the orthorhombic space group *P*2_1_2_1_2_1_. The sulfonamide NH_2_ donates an N—H⋯O hydrogen bond to both sides of the carbonyl group of the lactam acceptor in the synthon 1 catemer (Fig. 4 ▸ *a*). The hydrogen-bonded *C*(4) chain runs along the *a*-axis and in a corrugated sheet-like structure parallel to the (011) plane (Fig. 4 ▸, Fig. S1) and exhibits three-dimensional isostructurality in crystal packing.

#### 3.2.2. ClBSA–VLM, 4BrBSA–VLM (1:1)   

These two cocrystals have the catemer synthon and furthermore there is diversity in the two-dimensional packing patterns compared with the above set of five cocrystals. Both these structures are of the synthon 1 category even though they have different two-dimensional packing. The initial growth unit is the catemer hydrogen bond chain in these crystal structures. Sulfonamides and carboxamides form catemer synthon chains parallel to the *b*-axis (space group *C*2/*c*), which results in successive chain motifs (Fig. S1). The two-dimensional sheet arrangements of these isostructural cases are displayed in Fig. 4 ▸.

### 3.3. Synthon 2, dimer–cyclic ring   

#### 3.3.1. BSA–AZL cocrystal (1:1)   

**Figure 5:**
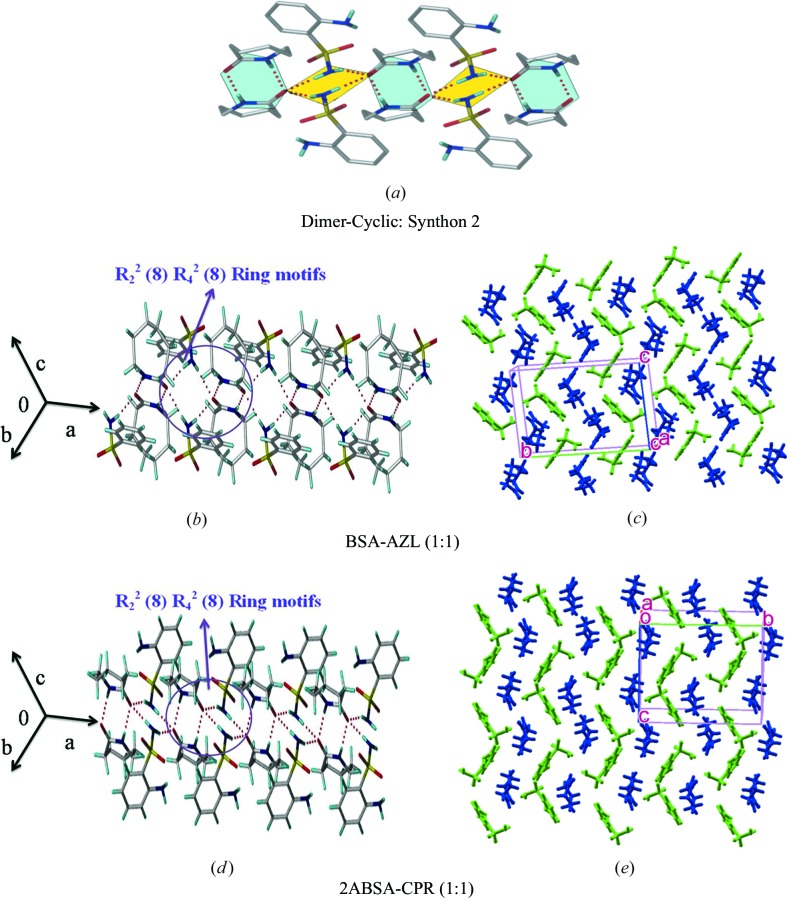
Dimer–cyclic synthon 2 in cocrystals BSA–AZL, 2ABSA–CPR and two-dimensional layer packing. Two-dimensional packing diagrams are drawn with the asymmetric unit showing benzene sulfonamides (in green) and lactams (in blue) (VLM, CPR).

The crystal structure was refined in the monoclinic space group *P*2_1_/*n*. Glide-related sulfonamide molecules are flanked between dimers of lactam through N—H⋯O (N1—H1*B*⋯O3: 2.12 Å, ∠158°; N1—H1*A*⋯O3: 2.03 Å, ∠158°) hydrogen bonds (sulfonamide NH donors) to give *R*_2_^2^(8)*R*_4_^2^(8) ring motif synthon 2 (Figs. 5 ▸ *a* and *b*), similar to that in N-oxide cocrystals (Goud *et al.*, 2011 ▸). The structural units extend along the *a*-axis in a one-dimensional pattern. The *meta* H atoms of BSA form C—H⋯O interactions with S=O along the *a*-axis (Fig. 5 ▸ *c*) resulting in corrugated layers of sulfonamide chains separated by coformer molecules (Fig. S2).

#### 3.3.2. ABSA–CPR cocrystal (1:1)   

This cocrystal is isostructural with BSA–AZL. The main synthon in 2ABSA–CPR is *R*_2_^2^(8)*R*_4_^2^(8) ring motifs along the *a*-axis (Fig. 5 ▸ *d*) together with corrugated wave-like layers (Figs. 5 ▸ *d* and *e*). The isostructurality is illustrated in Fig. S2.

### 3.4. Synthon 3, dimer–catemer   

#### 3.4.1. ClBSA–VLM cocrystal (1:1)   

**Figure 6:**
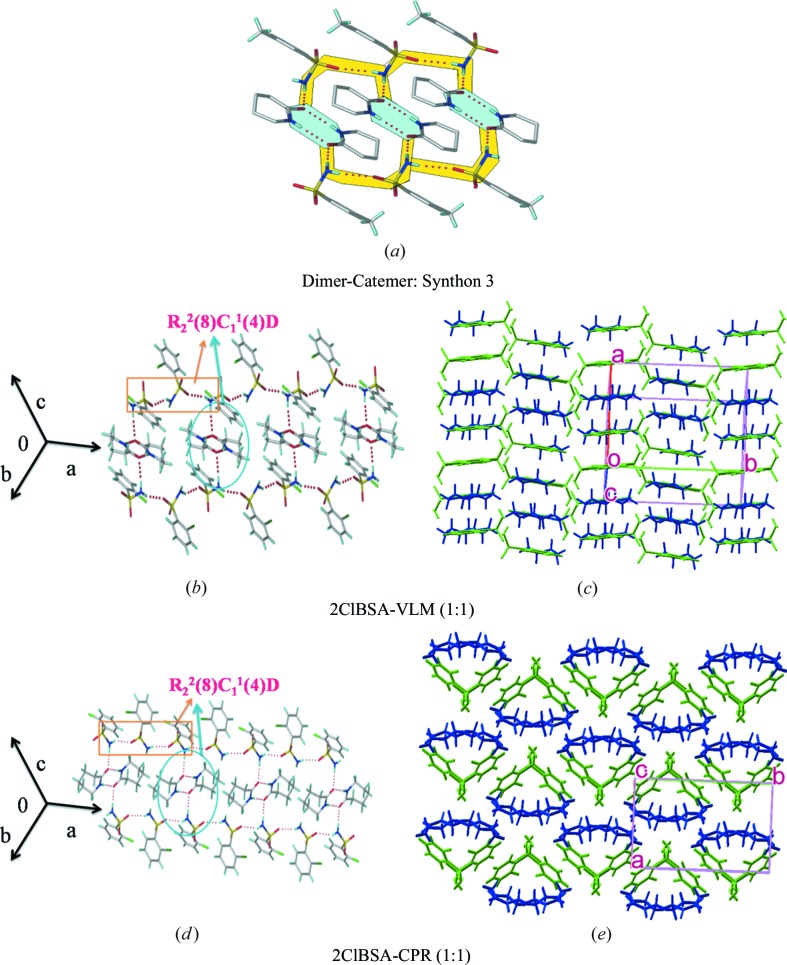

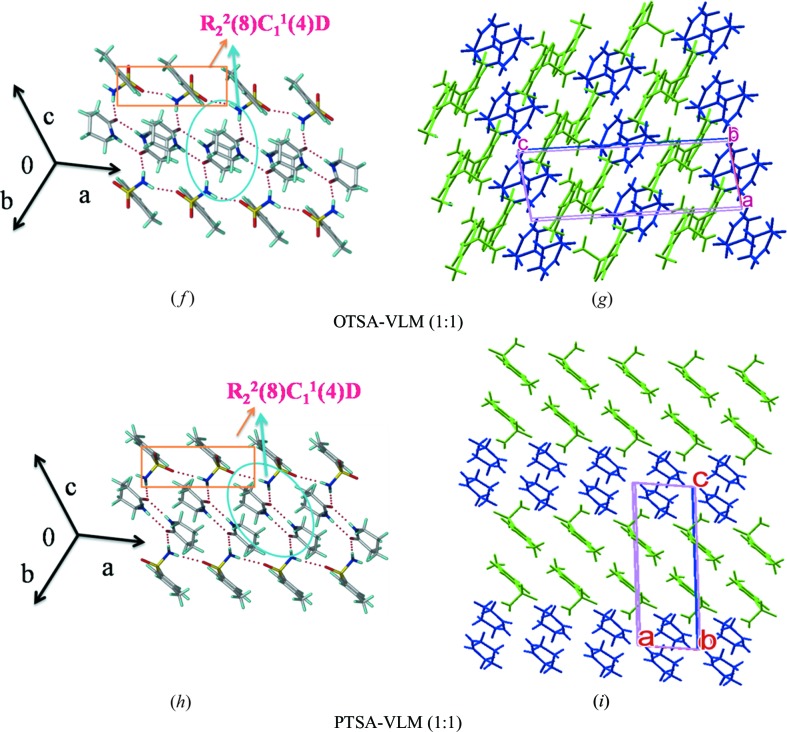
Dimer–catemer synthon 3 in cocrystals 2ClBSA–VLM, 2ClBSA–CPR, OTSA–VLM, PTSA–VLM and two-dimensional hydrogen bond motifs. Two-dimensional packing diagrams are drawn with the asymmetric unit showing benzene sulfonamides (in green) and lactams (in blue) (VLM, CPR).

Equimolar quantities of the components were ground and crystallized from EtOAc to give single crystals which were solved in the monoclinic space group *P*2_1_/*c*. Catemer chains connect glide-related 2ClBSA molecules that assemble *via* homodimers of VLM through N—H⋯O (N1—H1*A*⋯O3 = 2.03 Å, ∠169°) hydrogen bonds in synthon 3, or dimer–catemer synthon *R*_2_^2^(8)*C*_1_^1^(4)*D* (Figs. 6 ▸ *a* and *b*). In this synthon the coformer dimers are sandwiched between sulfonamide catemer chains. Halogen bonding (Cl⋯O, Cl⋯N) provides auxiliary support to the structure (Metrangolo *et al.*, 2005 ▸, 2008 ▸; Saha & Nangia, 2007 ▸; Desiraju, 1989 ▸; Mukherjee *et al.*, 2014 ▸). The catemer chains of 2ClBSA extend along the *c*-axis and homodimers of VLM connect adjacent chains of sulfonamides *via* C—H⋯O interactions to make two-dimensional stacks in the *ab*-plane (Fig. 6 ▸ *c*).

#### 3.4.2. ClBSA–CPR cocrystal (1:1)   

Cocrystal 2ClBSA–CPR is isostructural with 2ClBSA–VLM. Sulfonamide catemer chains are interlinked *via* discrete synthons to homodimers of CPR through N1—H1*A*⋯O3 hydrogen bonds (1.97 Å, ∠176°) to give synthon 3, dimer–catemer (Figs. 6 ▸ *a* and *d*). The homodimers of CPR are sandwiched between chains of sulfonamide chains. These patterns grow *via* C—H⋯O interactions to make interestingly parachute-like cone rings (Fig. 6 ▸ *e*).

#### 3.4.3. OTSA–VLM cocrystal (1:1)   

The OTSA molecule formed a cocrystal (monoclinic crystal system, *P*2_1_/*c* space group) with VLM homodimers (N2—H2*A*⋯O3 = 2.26 Å, ∠175°) *via* a discrete (*D* graph set) N—H⋯O (N1—H1*B*⋯O3 = 2.03 Å, ∠179°) synthon along the *c*-axis. Such dimers are sandwiched between screw-related sulfonamide chains, similar to two previous cocrystal structures (Fig. 6 ▸ *f*). Supportive C—H⋯O interactions make parallel stacks (Fig. 6 ▸ *g* and Fig. S3*a*).

#### 3.4.4. PTSA–VLM cocrystal (1:1)   

The crystal structure was solved in a triclinic crystal system with space group 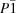. The basic supramolecular synthon of the catemer type is also present in this cocrystal (Fig. 6 ▸ *h*), but with different unit-cell parameters (Table 3 ▸). Sulfonamide molecules form catemer chain motifs above and below the VLM homodimers (N2—H2*A*⋯O3; H⋯O 2.15 Å, ∠176°; Fig. 6 ▸ *i*). The sandwich-type structure is sustained by inversion-related sulfonamide chains in *AABB*-type stacking (Fig. S3*b*).

### 3.5. Isostructural and isomorphous systems   

Two crystals are said to be isostructural if they have the same structure, but not necessarily the same unit-cell dimensions nor the same chemical composition, with a comparable variability in the atomic coordinates to that of the cell dimensions and chemical composition (IUCr, 2014 ▸). Isostructurality depicts the arrangement of different molecules in a similar way in the crystal structure, but not necessarily their unit-cell parameters (Fábián, Argay & Kálmán, 1999 ▸; Fábián & Kálmán, 1999 ▸, 2004 ▸; Kitaigorodsky, 1961 ▸). Certain substituents in the molecule can be replaced with others without altering the crystal packing as well as cell values and the space group (Brink & Kroese, 1952 ▸; Perutz, 1956 ▸; Kroon *et al.*, 1965 ▸; Sauer *et al.*, 1997 ▸; Dikundwar *et al.*, 2012 ▸). Such a functional group exchange leads to isostructural and isomorphous crystal structures (Berzelius, 1844 ▸; Melhado, 1980 ▸; Mitscherlich, 1822 ▸; Morrow, 1969 ▸). The recent literature on molecular cocrystals (Cinčić *et al.*, 2008*a* ▸,*b* ▸; Dubey & Desiraju, 2014 ▸) and pharmaceutical multi-component systems, *e.g.* lamotrigine and olanzapine cocrystals and salts, provide examples of isostructurality (Ebenezer *et al.*, 2011 ▸; Galcera *et al.*, 2012 ▸, 2013 ▸; Galcera & Molins, 2009 ▸; Clarke *et al.*, 2012 ▸; Thakuria & Nangia, 2013 ▸; Chitra *et al.*, 2012 ▸). The importance of isostructurality is that similar cocrystals can be designed depending on the geometry and shape and molecular composition of the starting materials. Isostructurality is also a useful guide in the crystal structure prediction of multi-component systems (Schmidt, 1971 ▸; Desiraju, 1989 ▸; Braga *et al.*, 1998 ▸; Desiraju *et al.*, 2011 ▸). Different guest molecules may be incorporated into the host lattice without substantially changing the crystal structure, *i.e.* isostructurality. The formation of isostructural cocrystals with the same synthon (isosynthon) and this study of sulfonamides with VLM, CPR shows how synthon similarity can lead to isostructural cocrystals (Fig. 3 ▸). There are four sets of isostructural cocrystals along with three types of synthons found in this set of cocrystals. Interestingly, a unique set of isostructural cocrystals shows isosynthons. Out of the 13 cocrystal structures in this study, four contain the dimer–catemer synthon, two result in the dimer–cyclic motif and seven gave the catemer synthon. Synthon 1 cocrystals exhibit two isostructural sets: set one of BSA–VLM, BSA–CPR, 4ClBSA–CPR, 4BrBSA–CPR, SNA–CPR and set two cocrystals 4ClBSA–CPR and 4BrBSA–CPR. These are three-dimensional isostructural systems and show isostructurality due to the Cl/Br/NH_2_ exchange (functional group) and VLM/CPR (homolog; Table 4 ▸). Further, the same trend continues for synthons 2 and 3 cocrystal sets also, *i.e.* isostructurality for Cl/Br and VLM/CPR. Furthermore, despite changes in molecular structures, the PXRD line patterns of synthon 1 cocrystals match quite well (Fig. S4) confirming their isomorphous nature.

Isostructurality was calculated on the basis of unit-cell parameters. Monoclinic and orthorhombic crystal structures show the unit-cell similarity index 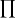 goes to zero (isostructurality) (see Table 2 ▸)
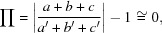
where *a*, *b*, *c* and *a*′, *b*′, *c*′ are orthogonalized lattice parameters of the related structures.

### 3.6. Classification of sulfonamide synthons   

**Figure 7:**
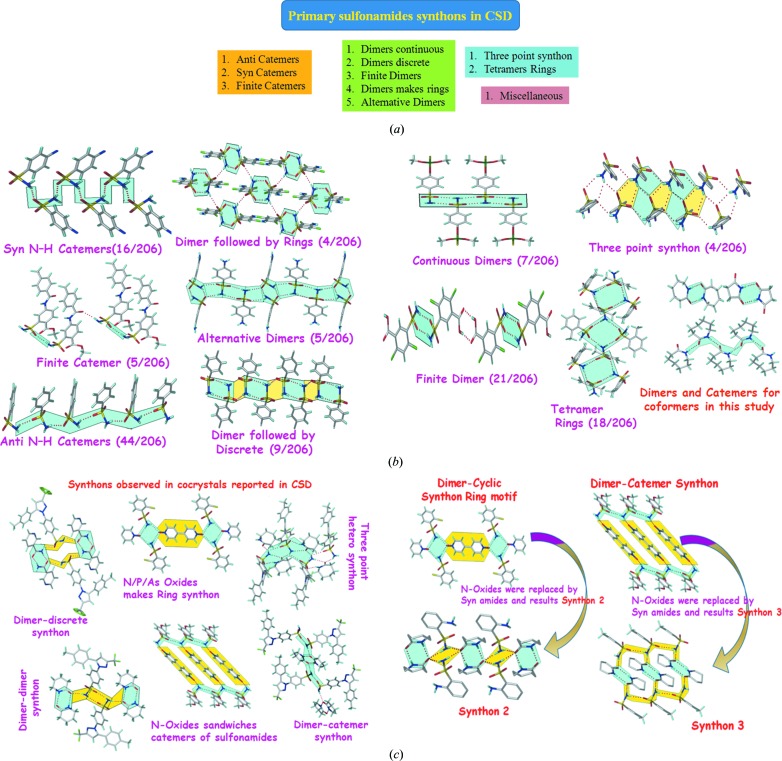
(*a*) Classification of primary sulfonamide synthons reported in CSD and (*b*) their hydrogen bonding and frequency. (*c*) Synthons in cocrystals of primary sulfonamides with N-oxides and amides. The latter analysis suggests that sulfonamide–N-oxide synthons may be replaced by *syn*-amides to give a new strategy for sulfonamide–carboxamide cocrystals.

A survey of the Cambridge Structural Database (CSD, Version 5.36, 1 November 2014 ▸ update; Allen, 2002 ▸) furnished 220 hits of primary sulfonamides (after eliminating hydrates, solvates, salts and duplicates) and 2160 hits of secondary sulfonamides (Table 5 ▸). These reported structures were analyzed to classify the known supramolecular synthons for sulfonamides and named as the *anti* catemer, *syn* catemer, finite catemer; continuous dimers, alternative dimers (Fig. 7 ▸ *b*), dimers making rings, finite dimers; tetramers, three point synthons, and finally a miscellaneous cluster of mixed motifs (Fig. 7 ▸, CSD refcodes are provided in Table S2). The presence of multiple donors/acceptors on the SO_2_NH_2_ group together with conformational flexibility (*syn*/*anti*) leads to many possible hydrogen bond synthons. In contrast, the *syn* amides are more predictable and show mainly dimer and to a lesser extent catemer synthons. The synthons in Fig. 7 ▸(*c*) suggest that the known heterosynthon between sulfonamide and N-oxide may be replaced by amide with the same graph set 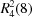 to provide a crystal engineering strategy for sulfonamide–carboxamide cocrystals.

A CSD search for the binary systems (cocrystals) furnished 33 hits for primary sulfonamides and 39 hits for secondary sulfonamides along with the starting materials of this study (Fig. S5). The almost equal numbers of primary and secondary sulfonamide cocrystals means that there are no steric issues with cocrystal assembly. Among the primary sulfonamide cocrystals, there are a few N—H⋯O hydrogen-bonded structures with amides, *e.g.* celecoxib-valerolactam trimorphs and nicotinamide cocrystals (see Fig. 7 ▸). Among the primary sulfonamide drugs, celecoxib, furosemide, acetazolamide and hydrochlorothiazide are notable for making cocrystals with amide coformers (Bolla *et al.*, 2014 ▸; Harriss *et al.*, 2014 ▸; Ueto *et al.*, 2012 ▸; Arenas-García *et al.*, 2010 ▸; Sanphui & Rajput, 2014 ▸; Remenar *et al.*, 2007 ▸), *e.g.* nicotinamide, isonicotinamide and picolinamide with different sulfonamide–amide syn­thons (see Fig. S9).

There are 2046 sulfonamides in the CSD but only 72 binary systems (cocrystals) in the CSD. The fewer number of sulfonamide cocrystals compared to say those for carboxylic acids and amides could be due to the enthalpy penalty for disrupting the strong sulfonamide homosynthon in the parent crystal structures with an even stronger hydrogen bond in the cocrystal. The activated oxygen acceptor of N-oxides, and to a lesser extent carboxamide functional groups, has been successfully used for sulfonamide cocrystals. The present study presents a crystal engineering approach to sulfonamide–carboxamide cocrystals analogous to the sulfonamide–pyridine-N-oxide heterosynthon.

### 3.7. Hirshfeld surface analysis   

**Figure 8:**
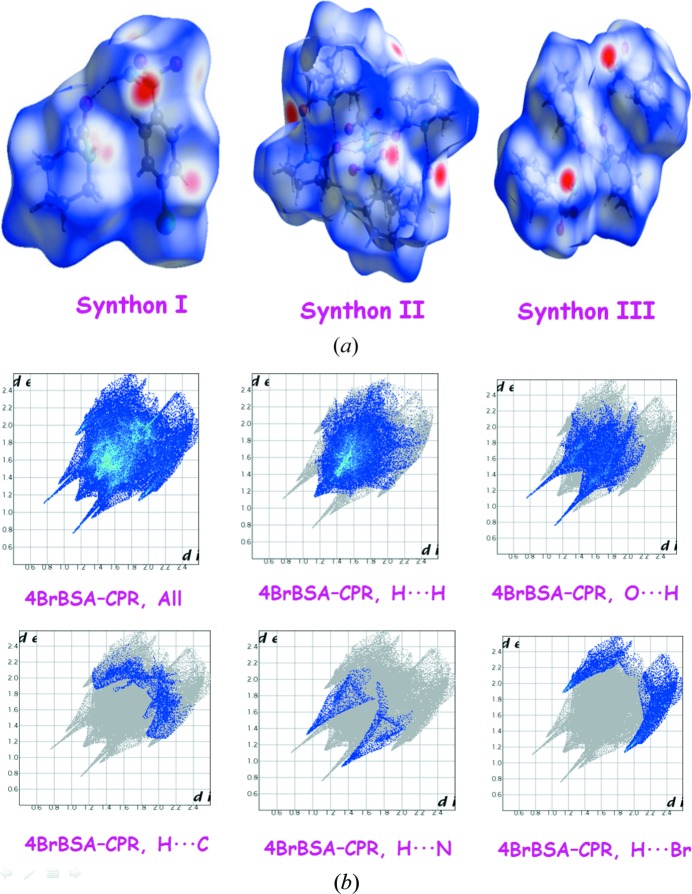
(*a*) Hirshfeld surfaces of the three types of the synthons present in sulfonamide–lactam cocrystals. (*b*) Two-dimensional fingerprint plots of the intermolecular contacts in the 4BrBSA–CPR cocrystal.

The Hirshfeld surface (using *Crystal Explorer*, Version 3.1, Hirshfeld, 1977 ▸; Hirshfeld & Mirsky, 1979 ▸; Kitaigorodsky, 1973 ▸; Vainshtein *et al.*, 1982 ▸; Spackman & Jayatilaka, 2009 ▸, McKinnon *et al.*, 1998 ▸) translates the electron density into molecular fragments and also volume around a molecule in a manner similar to the van der Waals surface, or an outer surface of the electron density in a crystal structure. The Hirshfeld surface is related to the molecule and the proximity of its nearest neighbors and this allows easy identification of characteristic strong and weak interactions throughout the structure. It explains the nature of intermolecular interactions within a crystal structure using a two-dimensional fingerprint plot consisting of spikes and wings. The 4BrBSA–VLM cocrystal two-dimensional finger plots with all types of interactions are shown in Fig. 8 ▸ as a representative of this class. The other binary systems are shown in Fig. S6. The strong spikes at 1.0–1.2 Å correspond to H⋯O interactions and the weak spikes between 1.2 and 1.4 Å for H⋯N hydrogen bonds. The other H⋯*X*, H⋯H, H⋯C interactions occur between 1.5 and 2.4 Å in the wings region. The strong H⋯O interaction is the major contributor in cocrystal structures (Fig. S7 and Table S3).

## 4. Conclusions   

A crystal engineering strategy is described for cocrystals of an otherwise less studied but pharmaceutically very important class of sulfonamide functional group. The binary systems of benzene sulfonamide–lactam exhibit three types of heterosynthons. The N—H donor of the sulfonamide forms a hydrogen bond with the C=O acceptor in different arrangements to result in synthon 1 of the catemer chain, synthon 2 as a dimer–cyclic motif and synthon 3 as a dimer–catemer. The classification of cocrystal structures in these synthon categories now offers a design element for sulfa drug cocrystals with GRAS coformers. Interestingly, isostructural pairs of cocrystals with isosynthons are observed in this study, which not only facilitates classification but also correlates with known cocrystal structures in the CSD, *e.g.* the novel sulfonamide–amide synthon analogous to the reported sulfonamide–N-oxide. The cocrystals of primary sulfonamides with GRAS coformers will provide an entry to the modification of sulfa drugs *via* pharmaceutical cocrystals.

## Supplementary Material

Crystal structure: contains datablock(s) global, 2ABSACPR, BSAAZL, 4ClBSACPR, SNACPR, 4BrBSACPR, OTSAVLM, 2ClBSACPR, BSACPR, 4ClBSAVLM, 4BrBSAVLM, PTSAVLM, 2ClBSAVLM, BSAVLM. DOI: 10.1107/S2052252515004960/zx5004sup1.cif

Structure factors: contains datablock(s) 2ABSACPR. DOI: 10.1107/S2052252515004960/zx50042ABSACPRsup2.hkl

Structure factors: contains datablock(s) BSAAZL. DOI: 10.1107/S2052252515004960/zx5004BSAAZLsup3.hkl

Structure factors: contains datablock(s) BSAVLM Structure factors: contains datablock(s) BSAVLM. DOI: 10.1107/S2052252515004960/zx5004BSAVLMsup14.hkl

Structure factors: contains datablock(s) SNACPR. DOI: 10.1107/S2052252515004960/zx5004SNACPRsup5.hkl

Structure factors: contains datablock(s) 4BrBSACPR. DOI: 10.1107/S2052252515004960/zx50044BrBSACPRsup6.hkl

Structure factors: contains datablock(s) OTSAVLM. DOI: 10.1107/S2052252515004960/zx5004OTSAVLMsup7.hkl

Structure factors: contains datablock(s) 2ClBSACPR. DOI: 10.1107/S2052252515004960/zx50042ClBSACPRsup8.hkl

Structure factors: contains datablock(s) BSACPR. DOI: 10.1107/S2052252515004960/zx5004BSACPRsup9.hkl

Structure factors: contains datablock(s) 4ClBSAVLM. DOI: 10.1107/S2052252515004960/zx50044ClBSAVLMsup10.hkl

Structure factors: contains datablock(s) 4BrBSAVLM. DOI: 10.1107/S2052252515004960/zx50044BrBSAVLMsup11.hkl

Structure factors: contains datablock(s) PTSAVLM. DOI: 10.1107/S2052252515004960/zx5004PTSAVLMsup12.hkl

Structure factors: contains datablock(s) 2ClBSAVLM. DOI: 10.1107/S2052252515004960/zx50042ClBSAVLMsup13.hkl

Structure factors: contains datablock(s) BSAVLM Structure factors: contains datablock(s) BSAVLM. DOI: 10.1107/S2052252515004960/zx5004BSAVLMsup14.hkl

Supporting tables and figures. DOI: 10.1107/S2052252515004960/zx5004sup15.pdf

CCDC references: 1039188, 1039189, 1039190, 1039191, 1039192, 1039193, 1039194, 1039195, 1039196, 1039197, 1039198, 1039199, 1039200, 1053703

## Figures and Tables

**Table 1:** Summary of characterization for sulfonamidelactam cocrystals 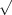
 = yes, = no.

		VLM (six member lactam)			CPR (seven member lactam)		
S. No.	Sulfonamides	Changes in IR	Changes in PXRD	Single crystal data	Changes in IR	Changes in PXRD	Single crystal data
1	BSA	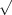	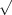	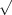	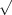	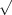	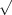
2	OTSA	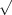	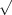	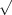	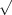	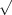	
3	PTSA	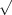	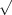	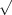	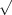	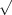	
4	SNA	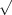	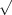	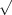	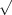	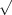	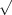
5	2ABSA	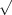	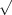		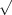	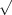	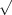
6	2ClBSA	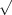	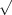	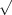	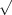	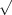	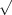
7	4ClBSA	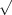	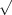	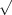	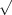	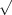	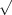
8	4BrBSA	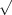	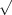	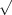	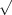	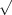	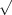

**Table 2:** Crystallographic data summary and classification of sulfonamidecarboxamide cocrystals and isostructurality (see Table 3 ▸ for full crystallographic data)

S. No.	Cocrystal	Cell parameters (*a*, *b*, *c*, in )	Crystal system	Synthon observed
1	BSAVLM	*a* = 7	Orthorhombic, *P*2_1_2_1_2_1_	
2	BSACPR			
3	4ClBSACPR	*b* = 1213		Synthon 1
4	4BrBSACPR	*c* = 1415		Catemer chain
5	SNACPR			
6	4ClBSAVLM	*a* = 25	Monoclinic, *C*2/*c*	
7	4BrBSAVLM	*b* = 7		
		*c* = 19		
8	2ABSACPR	*a* = 7	Monoclinic, *P*2_1_/*n*	Synthon 2
9	BSAAZL	*b* = 1617		DimerCyclic ring
		*c* = 1213		
10	2ClBSAVLM	*a* = 910	Monoclinic, *P*2_1_/*c*	Synthon 3
11	2ClBSACPR	*b* = 1314		DimerCatemer
		*c* = 10		
12	PTSAVLM	*a* = 5	Monoclinic, *P*2_1_/*n*	
		*b* = 16		
		*c* = 16		
13	OTSAVLM	*a* = 5	Triclinic, 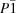	
		*b* = 8		
		*c* = 16		

**Table:** 

	Catemer synthon						
	BSAVLM	BSACPR	4ClBSACPR	4BrBSACPR	SNACPR	4ClBSAVLM	4BrBSAVLM
Empirical formula	C_6_H_7_NO_2_SC_5_H_9_NO	C_6_H_7_NO_2_SC_6_H_11_NO	C_6_H_6_ClNO_2_SC_6_H_11_NO	C_6_H_6_BrNO_2_SC_6_H_11_NO	C_6_H_8_N_2_O_2_SC_6_H_11_NO	C_6_H_6_ClNO_2_SC_5_H_9_NO	C_6_H_6_BrNO_2_SC_5_H_9_NO
Formula weight	256.33	270.35	304.80	349.25	285.37	290.77	335.22
Crystal system	Orthorhombic	Orthorhombic	Orthorhombic	Orthorhombic	Orthorhombic	Monoclinic	Monoclinic
Space group	*P*2_1_2_1_2_1_	*P*2_1_2_1_2_1_	*P*2_1_2_1_2_1_	*P*2_1_2_1_2_1_	*P*2_1_2_1_2_1_	*C*2/*c*	*C*2/*c*
*T* (K)	298(3)	298(3)	298(3)	298(3)	298(3)	298(3)	298(3)
*a* ()	7.1043(5)	7.0700(9)	7.1564(13)	7.156(3)	7.0957(6)	25.701(4)	25.914(3)
*b* ()	12.7937(10)	12.7624(13)	13.369(2)	13.538(5)	13.1280(13)	6.8096(4)	6.8687(9)
*c* ()	14.0302(16)	14.977(2)	15.276(3)	15.406(6)	15.3425(18)	19.177(3)	19.202(2)
()	90	90	90	90	90	90	90
()	90	90	90	90	90	127.40(2)	126.873(2)
()	90	90	90	90	90	90	90
*V* (^3^)	1275.2(2)	1351.4(3)	1461.5(4)	1492.5(10)	1429.2(3)	2666.2(9)	2734.2(6)
*D*_calc_ (gcm^3^)	1.335	1.329	1.385	1.554	1.326	1.449	1.629
(mm^1^)	0.253	0.242	0.409	2.899	0.235	0.445	3.161
range	3.5927.83	3.9626.72	2.8426.31	2.0026.38	2.6424.65	2.6626.31	1.9626.35
*Z*/*Z* ^1^	4/1	4/1	4/1	4/1	4/1	8/1	8/1
*h* range	4 +8	8 +7	8 +7	8 +8	7 +8	32 +30	32 +32
*k* range	15 +15	7 +15	12 +16	16 +16	8 + 15	8 +8	8 +8
*l* range	17 12	17 +16	12 +19	19 +19	18 15	23 +22	23 +23
Reflections collected	3791	3348	4403	15786	3692	5127	14029
Total reflections	2493	2197	2851	3029	2354	2732	2790
Observed reflections	2175	1595	1152	2405	1318	1677	2216
*R*_1_ [*I* > 2(*I*)]	0.0466	0.0777	0.0896	0.0398	0.0529	0.0595	0.0348
*wR*_2_ (all)	0.1201	0.1806	0.1141	0.0931	0.0796	0.1344	0.0939
Goodness-of-fit	1.059	1.231	0.968	1.025	0.901	1.067	1.029
X-ray diffractmeter	Oxford Gemini	Oxford Gemini	Oxford Gemini	Bruker Smart Apex	Oxford Gemini	Oxford Gemini	Bruker Smart Apex

**Table:** 

	Dimercyclic synthon ring		Dimercatemer synthon			
	2ABSACPR	BSAAZL	2ClBSAVLM	2ClBSACPR	OTSAVLM	PTSAVLM
Empirical formula	C_6_H_8_N_2_O_2_SC_6_H_11_NO	C_6_H_7_NO_2_SC_7_H_13_NO	C_6_H_6_ClNO_2_SC_5_H_9_NO	C_6_H_6_ClNO_2_SC_6_H_11_NO	C_7_H_9_NO_2_SC_5_ H_9_NO	C_7_H_9_NO_2_SC_5_ H_9_NO
Formula weight	285.37	284.36	290.77	304.80	270.35	270.35
Crystal system	Monoclinic	Monoclinic	Monoclinic	Monoclinic	Monoclinic	Triclinic
Space group	*P*2_1_/*n*	*P*2_1_/*n*	*P*2_1_/*c*	*P*2_1_/*c*	*P*2_1_/*n*	*P-*1
*T* (K)	29(3)	298(3)	298(3)	298(3)	298(3)	298(3)
*a* ()	7.2731(4)	7.3020(9)	10.521(2)	9.8782(6)	5.3367(6)	5.210(3)
*b* ()	15.9052(10)	17.189(2)	13.7661(12)	14.1720(6)	15.9206(17)	8.449(4)
*c* ()	12.7766(6)	12.2835(16)	10.3407(16)	10.8753(6)	16.070(3)	16.104(8)
()	90	90	90	90	90	82.894(8)
()	99.291(5)	106.760(2)	116.31(2)	112.850(7)	98.308(12)	82.798(8)
()	90	90	90	90	90	81.772(8)
*V* (^3^)	1458.61(14)	1476.3(3)	1342.5(4)	1403.00(15)	1351.0	692.005
*D*_calc_ (gcm^3^)	1.299	1.280	1.439	1.443	1.329	1.298
(mm^1^)	0.230	0.225	0.442	0.426	0.242	0.236
range	3.1228.72	2.9323.26	2.7326.37	2.8726.37	2.562.56	1.2826.37
*Z*/*Z* ^1^	4/1	4/1	4/1	4/1	4/1	2/1
*h* range	8 +8	8 +8	12 +13	12 +11	6 +6	6 +6
*k* range	16 +18	20 +20	15 +17	17 +16	19 +11	10 +10
*l* range	15 +14	14 +14	11 +12	13 + 10	14 +20	20 +19
Reflections collected	5525	13710	5058	5810	5072	7341
Total reflections	2488	2520	2731	2870	2759	2819
Observed reflections	1993	2154	2039	2483	1420	1969
*R*_1_ [*I* > 2(*I*)]	0.0383	0.0597	0.0435	0.0385	0.0645	0.0504
*wR*_2_ (all)	0.0995	0.1419	0.1144	0.1031	0.1183	0.1526
Goodness-of-fit	1.017	1.092	0.983	1.093	1.019	1.043
X-ray diffractmeter	Oxford Gemini	Bruker Smart Apex	Oxford Gemini	Oxford Gemini	Oxford Gemini	Bruker Smart Apex

**Table 4:** Unit-cell similarity index (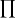) of cocrystals

Cocrystal†	Crystal system/space group	Cell values	Cell values summation	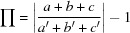
BSAVLM^*a*^	Orthorhombic, *P*2_1_2_1_2_1_	7.104, 12.793, 14.030	33.928	0.0253
BSACPR^*a*^		7.070, 12.762, 14.977	34.809	
4ClBSACPR^*a*^		7.156, 13.369, 15.276	35.801	0.0082
4BrBSACPR^*a*^		7.156, 13.538, 15.406	36.100	
SNACPR^*a*^		7.095, 13.128, 15.342	35.566	0.0147 (SNACPR, 4BrBSACPR)
4ClBSAVLM^*b*^	Monoclinic, * C*2/*c*	25.701, 6.809, 19.177	51.900	0.0016
4BrBSAVLM^*b*^		25.914, 6.8687, 19.202	51.984	
2ClBSAVLM^*c*^	Monoclinic, *P*2_1_/*c*	10.521, 13.7661, 10.340	34.627	0.0085
2ClBSACPR^*c*^		9.878, 14.172, 10.875	34.925	
2ABSACPR^*d*^		7.273, 15.905, 12.776	35.954	0.0223
BSAAZL^*d*^		7.302, 17.189, 12.2835	36.774	

† *a*, *b*, *c*, *d* are the different isomorphous systems as detailed in Table 1 ▸.

**Table 5:** CSD data on sulfonamides and their cocrystals Hydrates, solvates, salts and duplicates were removed in counting statistics.

Sulfonamides	CSD hits
No. of primary sulfonamides reported	220
No. of secondary sulfonamides reported	2160
No. of primary sulfonamides cocrystals reported	33
No. of secondary sulfonamides cocrystals reported	39
