# Supplementary material for: Modularity and three-dimensional isostructurality of novel synthons in sulfonamide–lactam cocrystals
Source: IUCrJ. 2015 May 1;2(Pt 4):389–401. doi: 10.1107/S2052252515004960 (PMC4491311; doi:10.1107/S2052252515004960)
Supplement: Supplementary file 14 [file m-02-00389-sup15.pdf]

IUCrJ

Volume 2 (2015)

Supporting information for article:

Modularity and three-dimensional isostructurality of novel synthons in sulfonamide–lactam cocrystals

Geetha Bolla, Sudhir Mittapalli and Ashwini Nangia

Table S1 Hydrogen bonding parameters in benzene sulfonamide cocrystals

Compound	Interactions	H···A/ Å	D···A/ Å	∠D–H···A/ º	Symmetry
2ABSA–CPR	N1–H1A···O3	2.18(2)	2.910(2)	159	1/2+x,1/2-y,1/2+z
	N1–H1B···O3	1.96(2)	2.855(3)	171	1/2-x,1/2+y,1/2-z
	N2–H2A···O2	2.28	3.108(2)	162	1/2+x,1/2-y,1/2+z
	N2–H2B···O1	2.18	2.892(2)	140	Intermolecular
	N3–H3A···O3	2.13	2.968(2)	165	1-x,-y,-z
2ClBSA–VLM	N1–H1A···O3	2.03	2.829(3)	169	a
	N1–H1B···Cl1	2.81	3.295(2)	118	Intermolecular
	N1–H1B···O1	2.16	2.922(2)	151	x,1/2-y,-1/2+z
	N2–H2A···O3	2.08	2.939(3)	177	1-x,-y,1-z
	C5–H5···O2	2.53	3.238(3)	133	-x,-1/2+y,1/2-z
2ClBSA–CPR	N1–H1A···O3	1.97	2.871(1)	176	a
	N1–H1B···O2	2.35	3.088(1)	151	x,1/2-y,1/2+z
	N2–H2A···O3	2.04	2.983(2)	175	1-x,-y,1-z
4BrBSA–VLM	N1–H1A···O3	2.06	2.866(4)	170	a
	N1–H1B···O3	2.00	2.879(3)	171	1/2-x,1/2+y,1/2-z
	N2–H2A···N1	2.50	3.172(4)	154	1/2-x,1/2+y,1/2-z
4BrBSA–CPR	N1–H1A···O3	1.96	2.887(5)	169	-x,1/2+y,1/2-z
	N1–H1B···O3	2.31	2.960(5)	159	1/2-x,-y,-1/2+z
	N2–H2A···N1	2.58	3.301(6)	144	1-x,-1/2+y,1/2-z
	C5–H5···O2	2.40	3.151(5)	138	-1+x,y,z
4ClBSA–VLM	N1–H1A···O3	2.08	2.881(4)	164	1/2-x,1/2+y,1/2-z
	N1–H1B···O3	2.04	2.855(5)	172	a
	N2–H2A···N1	2.36	3.153(5)	153	1/2-x,1/2+y,1/2-z
4ClBSA–CPR	N1–H1A···O3	2.19	2.908(1)	147	1-x,1/2+y,1/2-z
	N1–H1B···O3	1.89	2.850(1)	171	3/2-x,-y,-1/2+z
	N2–H2A···N1	2.56	3.285(2)	142	,-y,1/2+z
	C3–H3···O1	2.39	3.145(1)	138	1+x,y,z
BSA–VLM	N1–H1A···O3	2.05	2.890(1)	164	1/2+x,1/2-y,1/2+z
	N1–H1B···O3	2.12	2.908(2)	161	1/2-x,-y,-1/2+z
	N2–H2A···N1	2.48	3.270(1)	152	1+x,y,z
BSA–CPR	N1–H1A···O3	2.04	2.871(2)	159	1/2-x,1-y, ½+z

	N1–H1B···O3	2.10	2.859(1)	170	1-x,1/2+y, 3/2-z
	N2–H2A···N1	2.49	3.252(1)	148	1-x,-1/2+y,1/2-z
	C2–H2···O3	2.69	3.406(2)	134	-x,-1/2+y,1/2-z
BSA–AZL	N1–H1A···O3	2.12	2.879(3)	158	1/2+x,1/2-y,1/2+z
	N1–H1B···O3	2.03	2.905(4)	169	1/2-x,-1/2+y,1/2-z
	N2–H2A···O3	2.18	2.999(3)	168	1-x,1-y,-z
OTSA–VLM	N1–H1A···O3	2.03	2.857(2)	161	1/2-x,-1/2+y,1/2-z
	N1–H1B···O1	2.10	2.988(2)	157	1+x,y,z
	N2–H2A···O3	2.09	2.948(1)	175	1-x,1-y,1-z
PTSA–VLM	N1–H1A···O1	2.26	3.015(3)	163	1+x,y,z
	N1–H1B···O3	2.03	2.886(3)	179	a
	N2–H2A···O3	2.15	2.904(3)	176	-x,1-y,-z
SNA–CPR	N1–H1A···O3	2.16	2.902(5)	150	1/2-x,-y,-1/2+z
	N1–H1B···O3	1.96	2.845(5)	164	-x,1/2+y,1/2-z
	N2–H2A···O1	2.41	3.264(5)	166	1-x,-1/2+y,1/2-z
	N2–H2B···O2	2.26	2.998(5)	142	-x,-1/2+y,1/2-z
	N3–H3A···N1	2.46	3.240(5)	150	1-x,-1/2+y,1/2-z
	C3–H3···O2	2.57	3.482(5)	168	1-x,-1/2+y,1/2-z

[a] Molecules in the same asymmetric unit.

Compound	Interactions	H···A/ Å	D···A/ Å	∠D–H···A/ º	Symmetry
2ABSA–CPR	N1–H1A···O3	2.18(2)	2.910(2)	159	1/2+x,1/2-y,1/2+z
	N1–H1B···O3	1.96(2)	2.855(3)	171	1/2-x,1/2+y,1/2-z
	N2–H2A···O2	2.28	3.108(2)	162	1/2+x,1/2-y,1/2+z
	N2–H2B···O1	2.18	2.892(2)	140	Intermolecular
	N3–H3A···O3	2.13	2.968(2)	165	1-x,-y,-z
2ClBSA–VLM	N1–H1A···O3	2.03	2.829(3)	169	a
	N1–H1B···Cl1	2.81	3.295(2)	118	Intermolecular
	N1–H1B···O1	2.16	2.922(2)	151	x,1/2-y,-1/2+z
	N2–H2A···O3	2.08	2.939(3)	177	1-x,-y,1-z
	C5–H5···O2	2.53	3.238(3)	133	-x,-1/2+y,1/2-z
2ClBSA–CPR	N1–H1A···O3	1.97	2.871(1)	176	a
	N1–H1B···O2	2.35	3.088(1)	151	x,1/2-y,1/2+z
	N2–H2A···O3	2.04	2.983(2)	175	1-x,-y,1-z
4BrBSA–VLM	N1–H1A···O3	2.06	2.866(4)	170	a
	N1–H1B···O3	2.00	2.879(3)	171	1/2-x,1/2+y,1/2-z
	N2–H2A···N1	2.50	3.172(4)	154	1/2-x,1/2+y,1/2-z
4BrBSA–CPR	N1–H1A···O3	1.96	2.887(5)	169	-x,1/2+y,1/2-z
	N1–H1B···O3	2.31	2.960(5)	159	1/2-x,-y,-1/2+z
	N2–H2A···N1	2.58	3.301(6)	144	1-x,-1/2+y,1/2-z
	C5–H5···O2	2.40	3.151(5)	138	-1+x,y,z
4ClBSA–VLM	N1–H1A···O3	2.08	2.881(4)	164	1/2-x,1/2+y,1/2-z
	N1–H1B···O3	2.04	2.855(5)	172	a
	N2–H2A···N1	2.36	3.153(5)	153	1/2-x,1/2+y,1/2-z
4ClBSA–CPR	N1–H1A···O3	2.19	2.908(1)	147	1-x,1/2+y,1/2-z
	N1–H1B···O3	1.89	2.850(1)	171	3/2-x,-y,-1/2+z
	N2–H2A···N1	2.56	3.285(2)	142	,-y,1/2+z
	C3–H3···O1	2.39	3.145(1)	138	1+x,y,z
BSA–VLM	N1–H1A···O3	2.05	2.890(1)	164	1/2+x,1/2-y,1/2+z
	N1–H1B···O3	2.12	2.908(2)	161	1/2-x,-y,-1/2+z
	N2–H2A···N1	2.48	3.270(1)	152	1+x,y,z

BSA–CPR	N1–H1A···O3	2.04	2.871(2)	159	1/2-x,1-y, ½+z
	N1–H1B···O3	2.10	2.859(1)	170	1-x,1/2+y, 3/2-z
	N2–H2A···N1	2.49	3.252(1)	148	1-x,-1/2+y,1/2-z
	C2–H2···O3	2.69	3.406(2)	134	-x,-1/2+y,1/2-z
BSA–AZL	N1–H1A···O3	2.12	2.879(3)	158	1/2+x,1/2-y,1/2+z
	N1–H1B···O3	2.03	2.905(4)	169	1/2-x,-1/2+y,1/2-z
	N2–H2A···O3	2.18	2.999(3)	168	1-x,1-y,-z
OTSA–VLM	N1–H1A···O3	2.03	2.857(2)	161	1/2-x,-1/2+y,1/2-z
	N1–H1B···O1	2.10	2.988(2)	157	1+x,y,z
	N2–H2A···O3	2.09	2.948(1)	175	1-x,1-y,1-z
PTSA–VLM	N1–H1A···O1	2.26	3.015(3)	163	1+x,y,z
	N1–H1B···O3	2.03	2.886(3)	179	a
	N2–H2A···O3	2.15	2.904(3)	176	-x,1-y,-z
SNA–CPR	N1–H1A···O3	2.16	2.902(5)	150	1/2-x,-y,-1/2+z
	N1–H1B···O3	1.96	2.845(5)	164	-x,1/2+y,1/2-z
	N2–H2A···O1	2.41	3.264(5)	166	1-x,-1/2+y,1/2-z
	N2–H2B···O2	2.26	2.998(5)	142	-x,-1/2+y,1/2-z
	N3–H3A···N1	2.46	3.240(5)	150	1-x,-1/2+y,1/2-z
	C3–H3···O2	2.57	3.482(5)	168	1-x,-1/2+y,1/2-z

[a] Molecules/ions in the same asymmetric unit.

Table S2 Classification of primary sulfonamides based on synthons present and their CSD REFCODEs

**Sulfonamides (220)**							
**Alternative dimers (5)**							
PECTOI	RIBQID	VUSMEC	WOBHUR	ZEBYEM			
**Anti Catemer (50)**							
ADAGUI	AFIMEJ	AMEVAP	AZADAH	AZAGUE	BAPYIB	BAPYIB	BIZPIK
BIZPOQ	BSBTHP	AZACOU	CIVBAK10	DIBBUL	DUCVED	EWUHAF	EXICIX
EXICIX	FAVWIJ	FAXZIO	GEQYIK	GODRUM	HCSBTZ	ICUTOR	JEFNOY
JIGCUX	KIKCUD	KUSVEZ	KUZWIM	MESXIR	PIMZAO	POHYER	PUFHUU
QUYKOL	QUYRIM	SODGID	SULAMD03	SULTAM	SULTAM01	ULIDOJ	VUXPUZ
WEPMAH	ZEBYOW	BATNOB	CAFVUA	COYVER	GOMSAE	JEFPAM	LAWKUP
NIWPAL	OCAMUB						
**Dimer discrete (9)**							
AZADEL	GUFQED	IDIMOY	JEFNAK	KIXHEF	RIHGOF	TIGLOL	VABFAH
XAVKUB							
**Continuous dimers (7)**							
AFONAM	GUCMEW	JABJAY	PERQOT	SETHEE	USOBAG	TIYXII	
**Finite catemers (7)**							
IFAXAX	JAWHOF	SEQJUT	VOHZIC	ZAPJAD	ENIROI	GOFYIL	
**Finite dimers (21)**							
CIKJAI	CSBSMP	EFOFAH	EVAGUE	GODSAT	KIXHEF	LAPNAT	LAPPEZ
LISKUU	MEWXAP	MUWCUD	SEBPAQ	SEQKEE	WACJIT	WAWNAK	XETFAF
ZAHBEP	ATDZSA	CEFMUX	PODPUU	LISLAB			
**Rings forms dimers (4)**							
AMAVIT	AZADIP	JEFPIU	PODQEF				
**Syn Catemers (16)**							
CIKSIA	CUDDEL	ETOMUV	GAQBUX	HUCSUU	JEFMUD	JEFNIS	LOMWIT
PAVBUL	PODQAB	PUMPOD	PUMPUJ	QUYTIO	WAWMUD	NIGNEY	MOKMIJ
**Tetramer rings (21)**							

BUDYEF	JABJEC	JEFMAJ	JEFNEO	JEFNUE	JEFPOA	KIFZUV	KUSVEZ
MESXUD	MSULPH01	MUBVOV	NIGNEY	PERQEJ	QISQOZ	TIBPAW	UJIBAR
WEBWUV	ZZZULS01	DOLDIT	EXICOD	HUFVOU			
**Three point synthons (4)**							
ADAGUI	FIYYES	FOGVIG01	RIFVIM				
**Miscellaneous (76)**							
ADILOP	AZAMEU	BIXMUR	BUFMAR	CADTEG	CANWIX	CBSTHP	CIKJAI
CIKSIA	CIMKOA	CSBSMP	DOSDOE	EFOFAH	EVAGUE	EVAWAA	FUZXUU
GEVBAL	GODSAT	GOMKUP	HABSOS	HILRAW	HSLSTZ	IFAFAX	IPSULF
JAWHOF	JAZVEL10	JAZVIP10	JAZVOV10	JAZZOZ10	JEFPEQ	KEFROC	KIXHEF
KIXPIQ	KUVFIR	LAPNAT	LAPPEZ	LEFFAF	LESZUG	LISKUU	MUWCUD
PEDBOR	PEHWEF	PILPIK	PODQAB	POWBUZ	PUMPOD	PYMSBZ11	QAKXUX
SEBPAQ	SEQJUT	SEQKEE	SODGID	SOJNEK	SULPIA10	TELWAI	UJIBEV
UJIBIZ	UJIBOF	VAWMOV	VOHZIC	VUXPUZ	WACJIT	WAWNAK	WIGXIU
WIXGIV	XETFAF	YIKZUP	ZAHBEP	ZAPJAD	ZEBYOW	XAVKUB01	XAVKUB
DOPVIP	GOMRUX	MESXOX	NARVUZ				

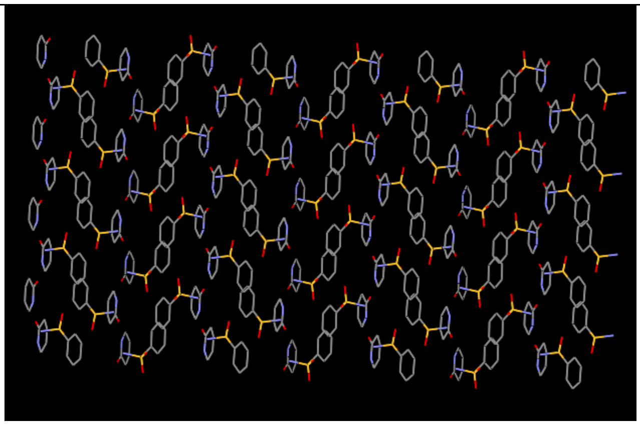

BSA–VLM

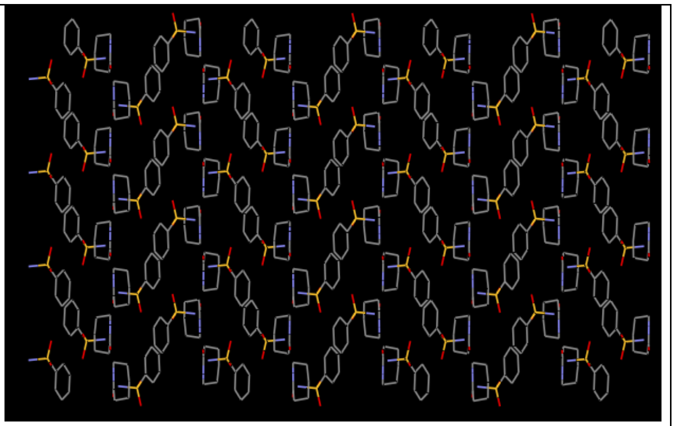

BSA–CPR

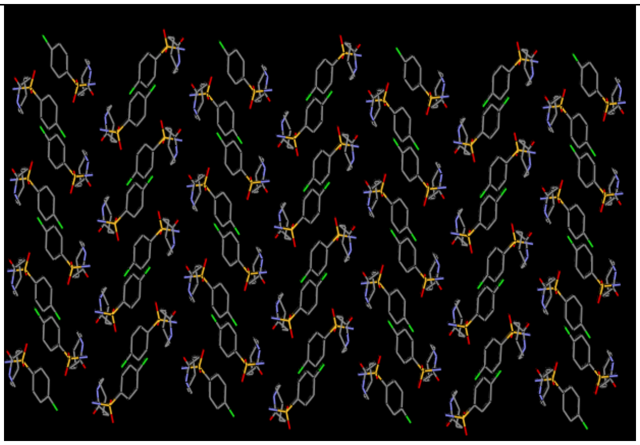

4ClBSA–CPR

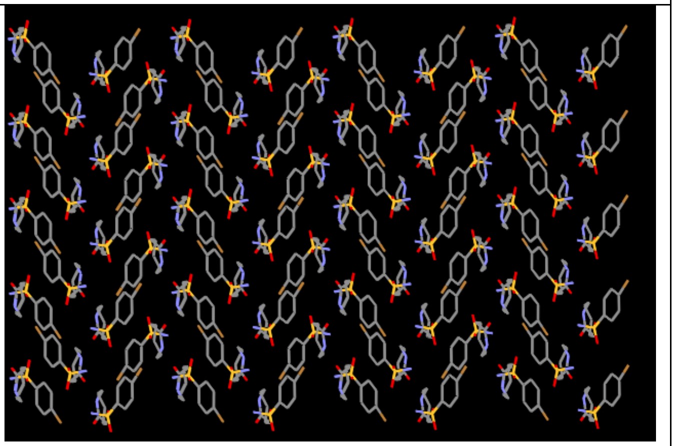

4BrBSA–CPR

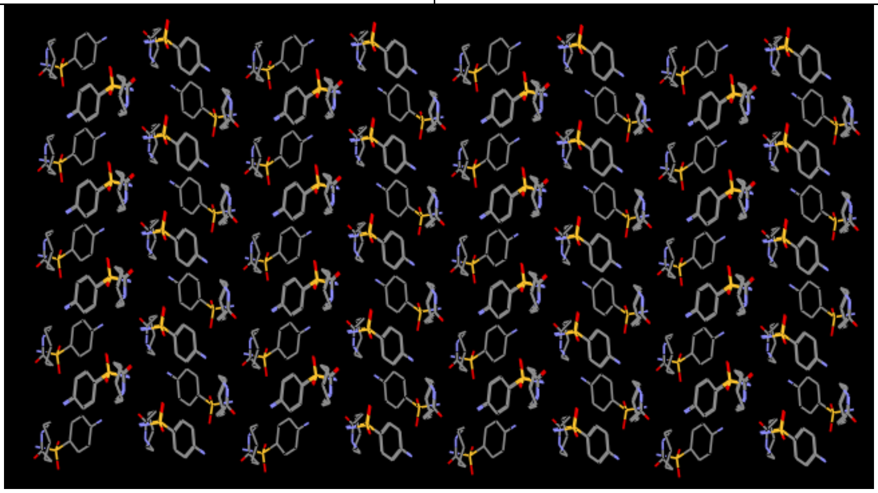

SNA–CPR

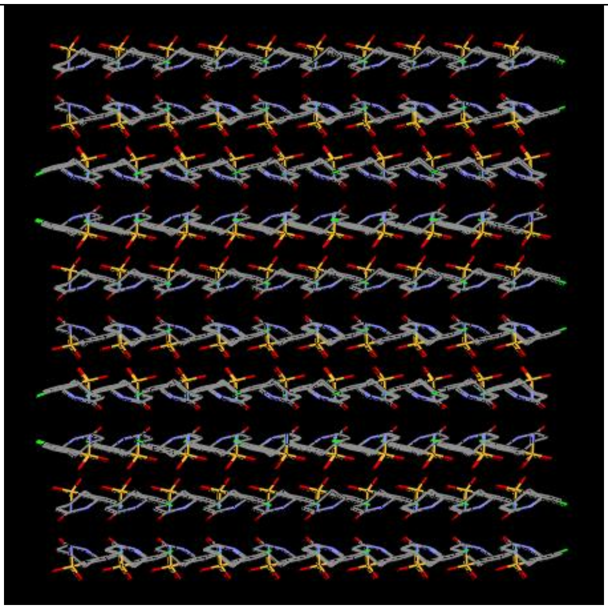

4ClBSA–VLM

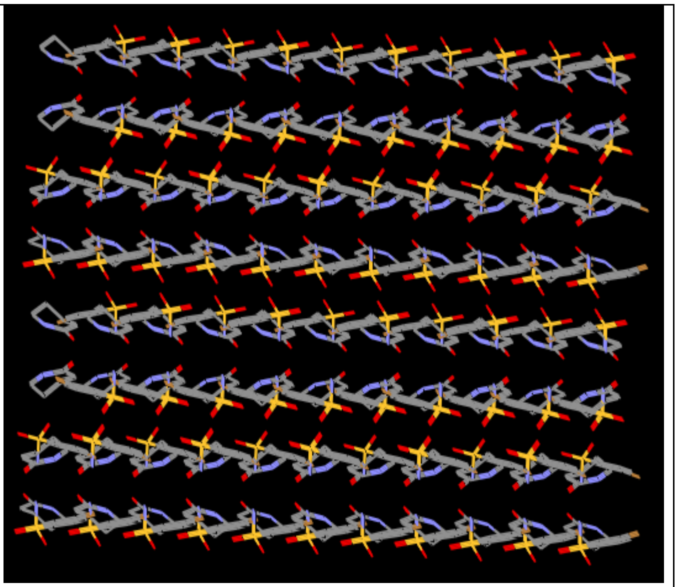

4BrBSA–VLM

Figure S1 2D sheet like structure propagation of the catemer chain Synthon 1 in cocrystal structures.

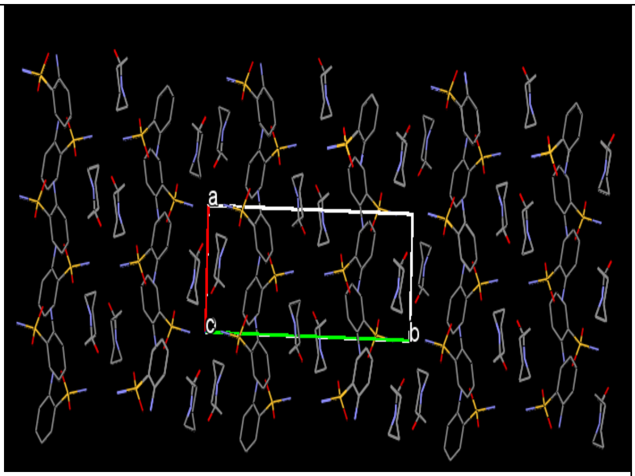

(a) 2ABSA–CPR

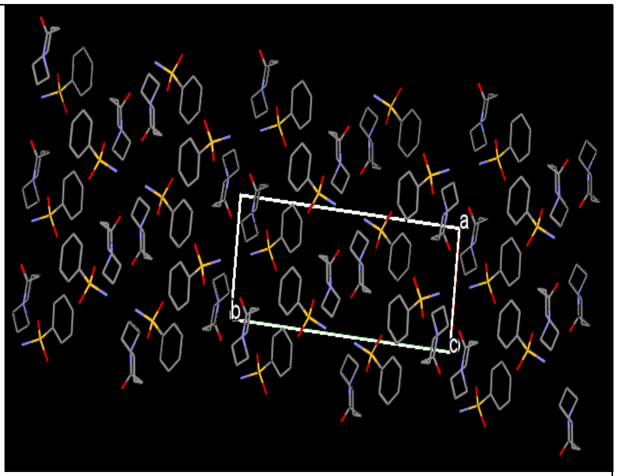

(b) BSA–AZL

Figure S2 Head to face packing thorugh dimer-cyclic ring motif Synthon 2 in cocrystals 2ABSA–CPR and BSA–AZL.

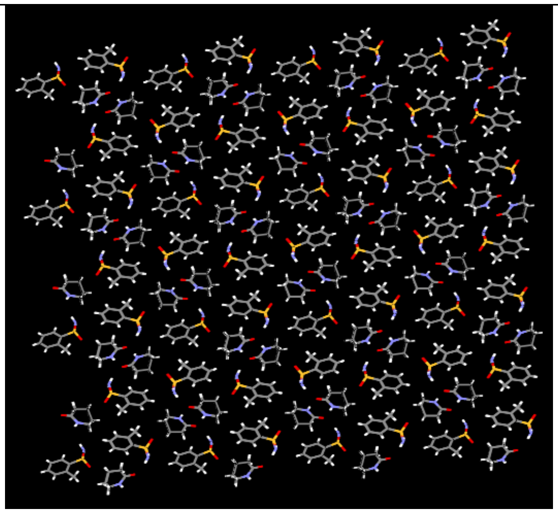

(a) OTSA–VLM

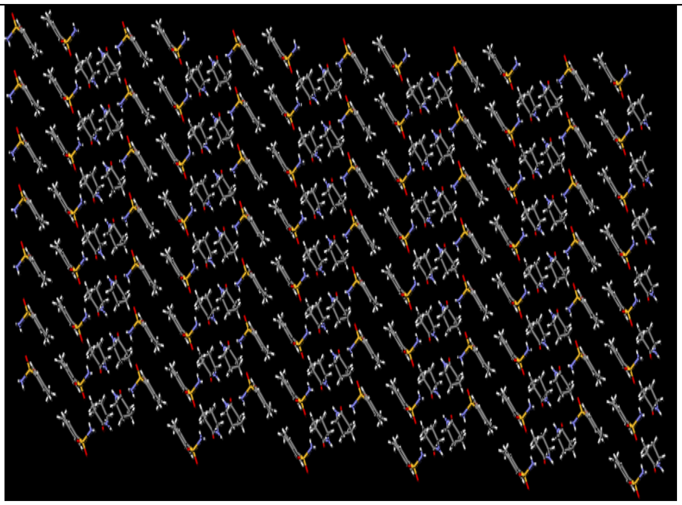

(b) PTSA–VLM

Figure S3 2D packing of OTSA–VLM and PTSA–VLM cocrystals in the *bc*-plane.

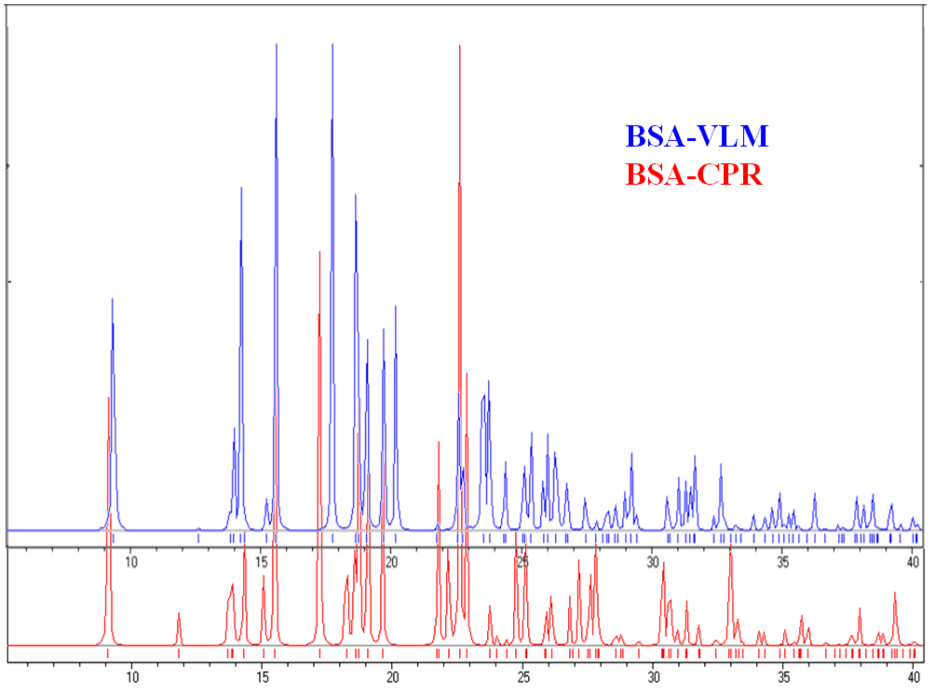

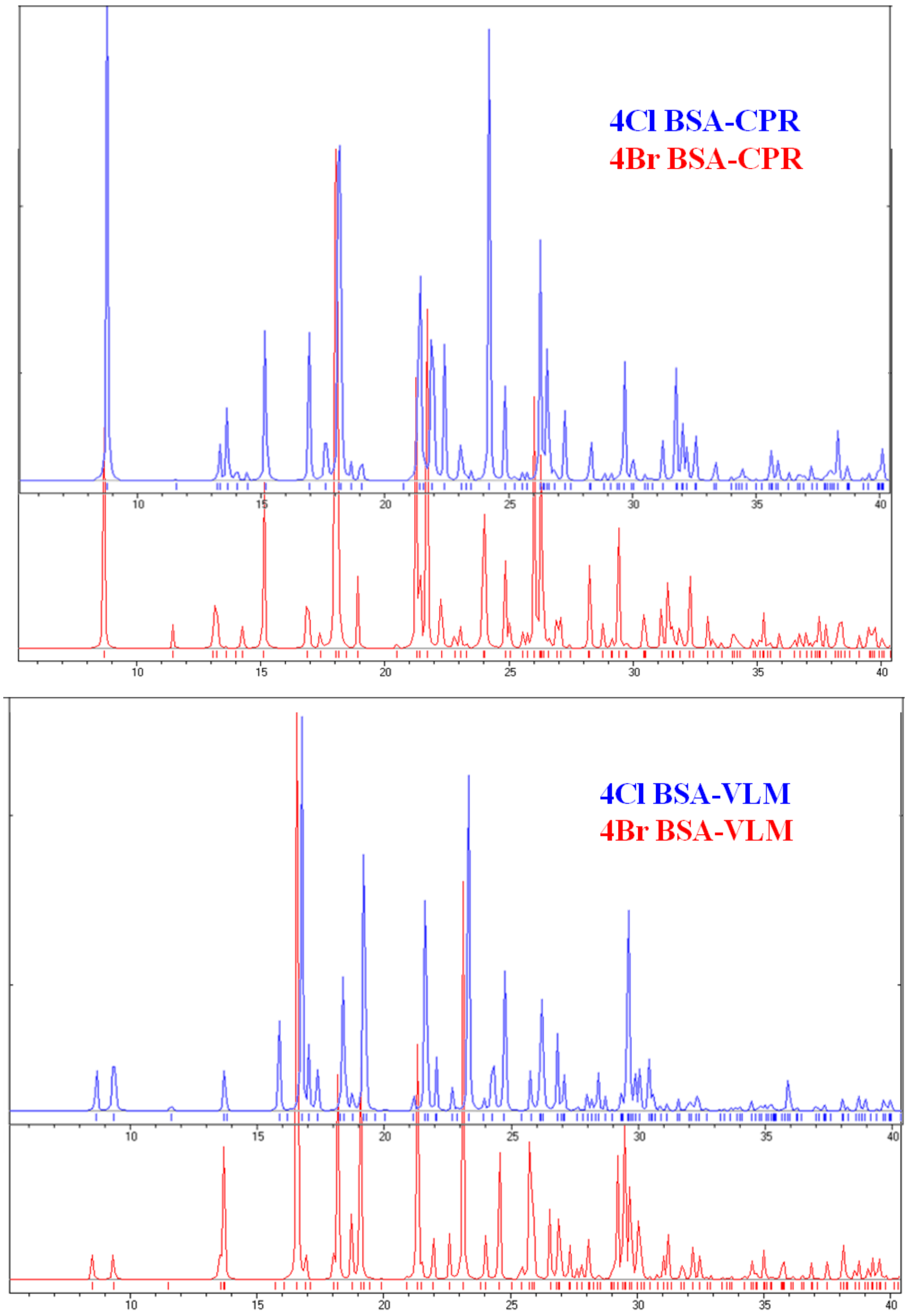

Figure S4 Powder X-ray diffraction patterns of the 3D isostructural catemer synthon cocrystals pairs. The near identical PXRD lines support isostructurility and isomorphous nature of the structures.

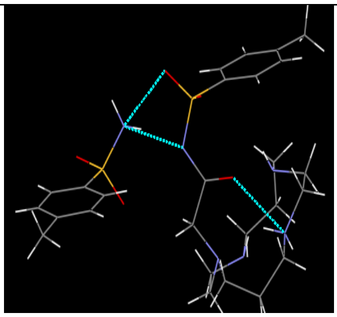<br>AQUFEY<br>N–H···O	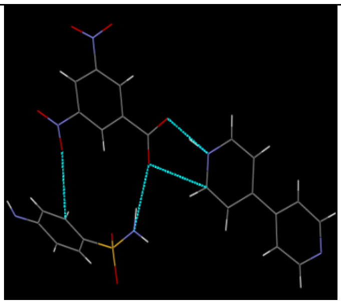<br>BEYZEM<br>N–H···O	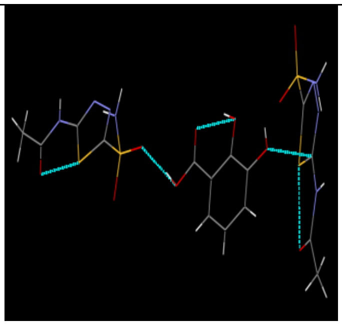<br>DATFAH<br>N–H···O	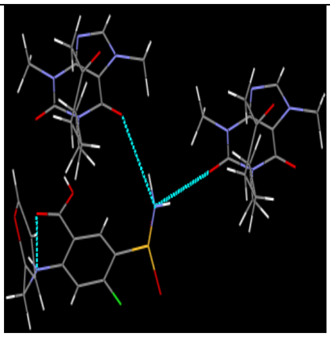<br>FEFYAS<br>N–H···O
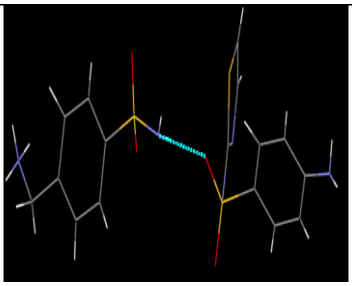<br>HSLSTZ<br>N–H···O	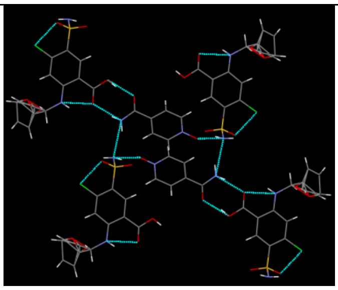<br>IWERIM<br>SO_2NH_2···N-oxide	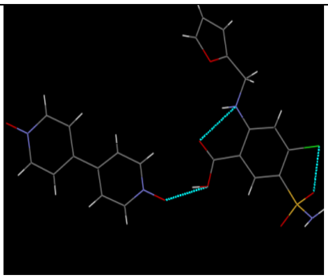<br>IWERUY<br>SO_2NH_2···N-oxide	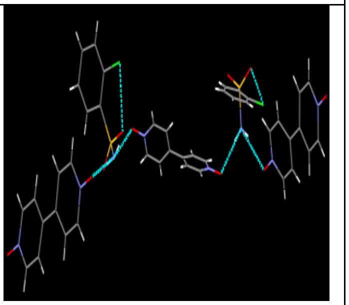<br>IWERAE<br>SO_2NH_2···N-oxide
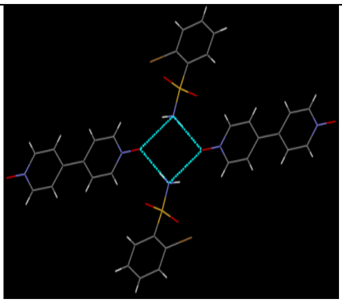<br>IWEQEX<br>SO_2NH_2···N-oxide	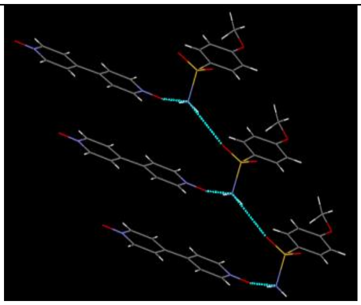<br>IWEREI<br>SO_2NH_2···N-oxide	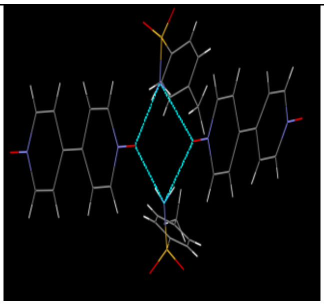<br>IWEROS<br>SO_2NH_2···N-oxide	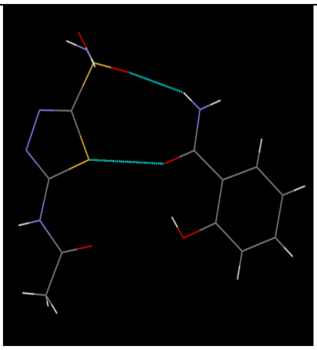<br>DATFEL<br>N–H···O
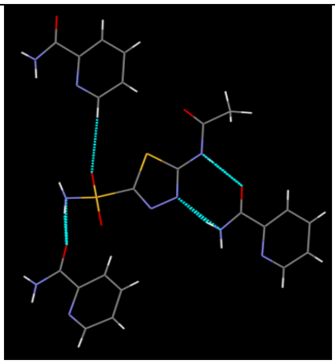	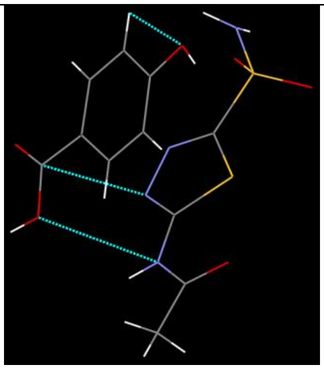	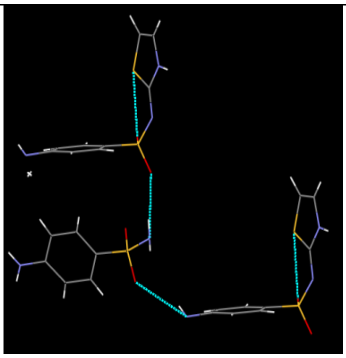	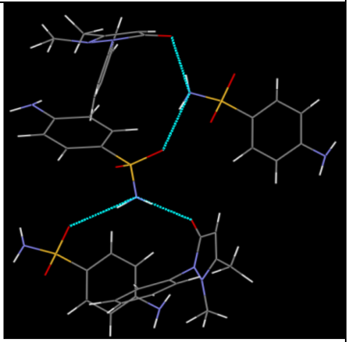<br>SANAPY

DATFIP<br>N–H···O	RUYGIC<br>N–H···O	STHSAM01<br>N–H···O	N–H···O
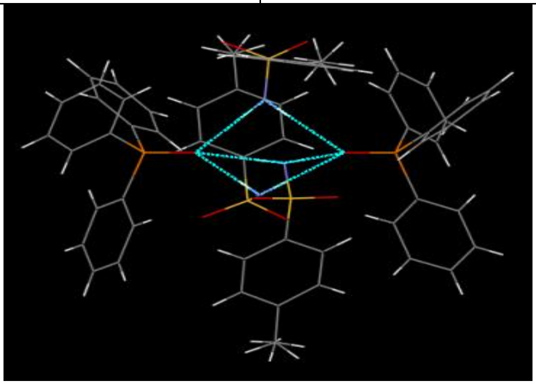<br>JACWEL<br>SO_2NH_2···P-oxide		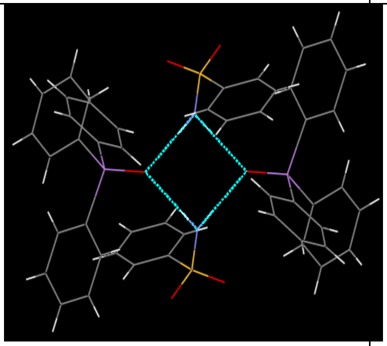<br>JAYCAN<br>SO_2NH_2···As-oxide	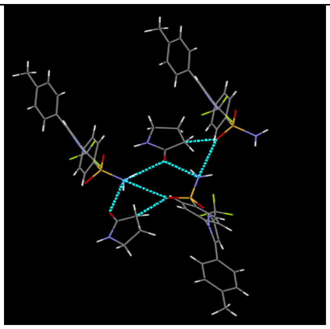<br>LIQPUY<br>N–H···O
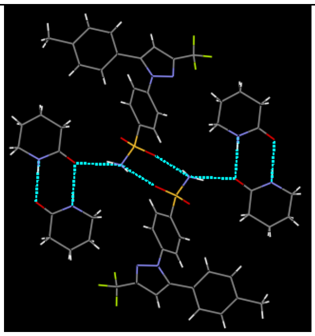<br>LIQQIN<br>N–H···O	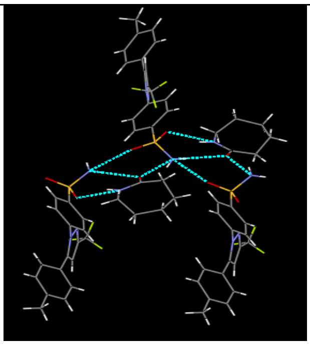<br>LIQQEJ<br>N–H···O	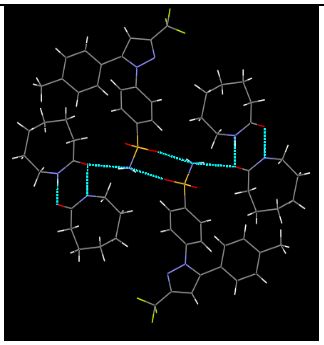<br>LIQQAF<br>N–H···O	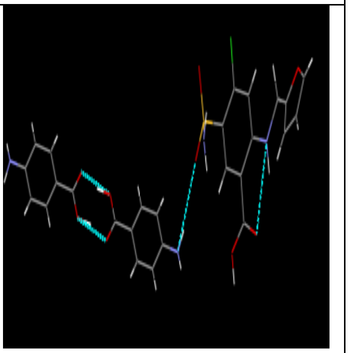<br>LOFLAV<br>N–H···O
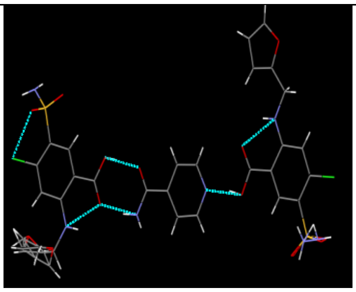<br>LOFLEZ<br>N–H···O	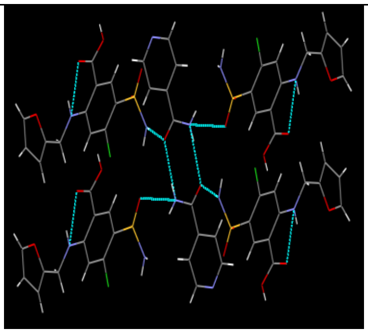<br>LOFLID<br>N–H···O	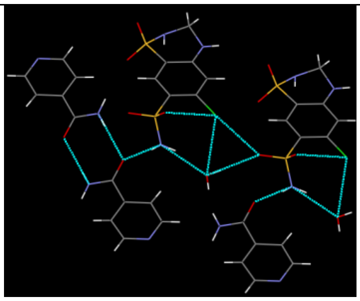<br>MUPPIX<br>N–H···O	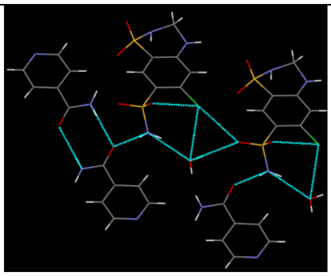<br>NARWAG<br>N–H···O

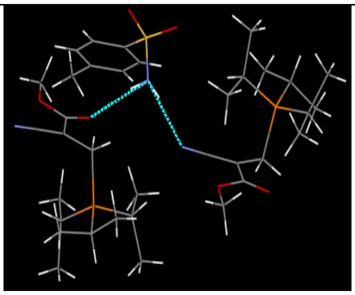<br>NETDIZ<br>N–H⋯O^{-}	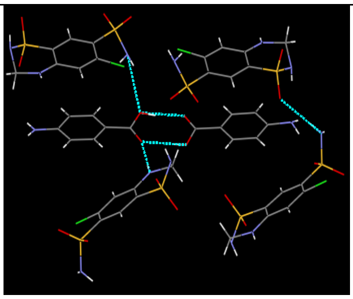<br>PIRLUZ<br>N–H⋯O	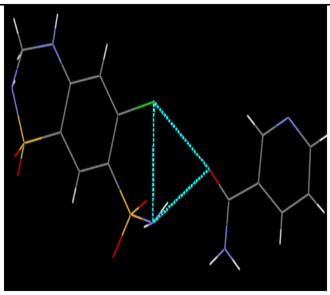<br>PIRXUL<br>N–H⋯O	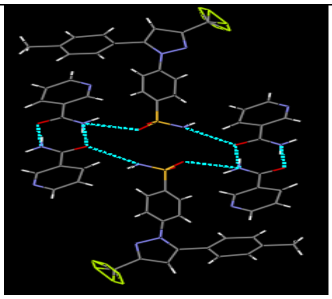<br>VIGDAR<br>N–H⋯O
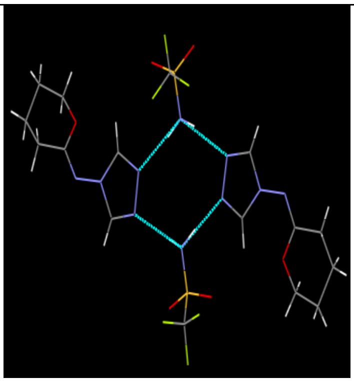<br>VOMMUF<br>N–H⋯N	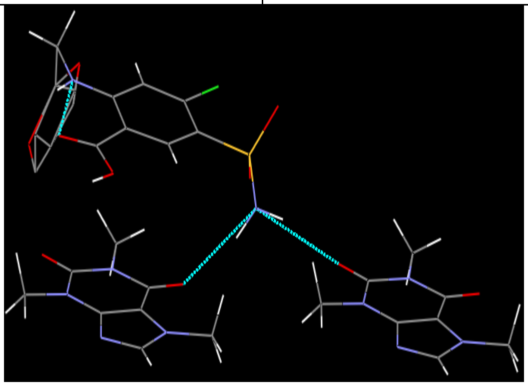<br>XAVTEV<br>N–H⋯O		

(a)

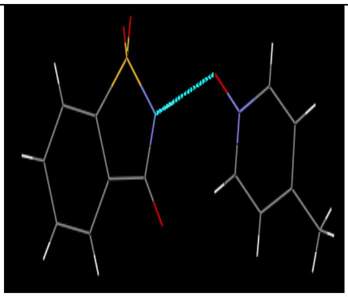<br>ADECIW<br>SO_2NH_2⋯N-oxide	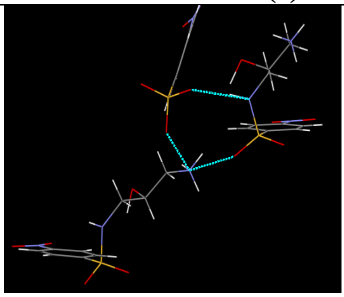<br>AGOKUC<br>N–H⋯O	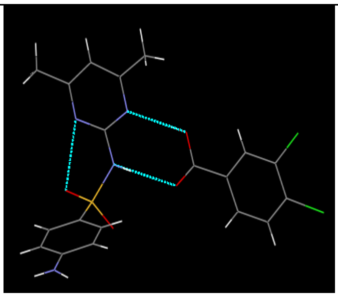<br>EXANEX<br>2-amino-Py⋯COOH	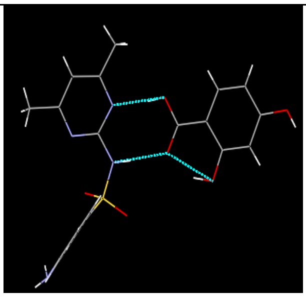<br>EXANAT<br>2-amino-Py⋯COOH
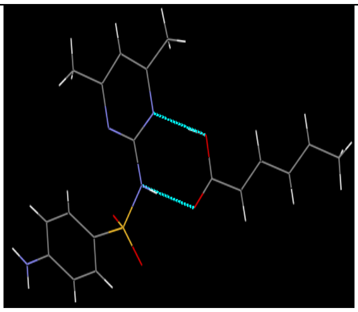<br>EXANIB	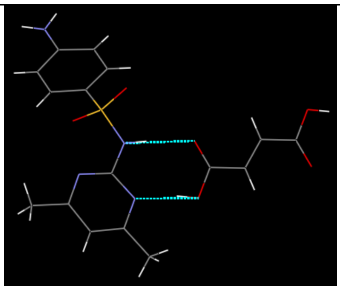<br>EXANOH	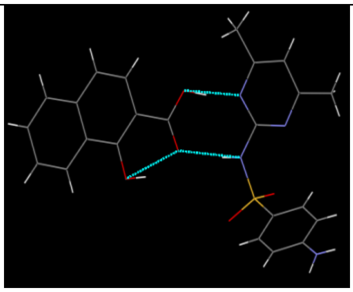<br>EXANUN	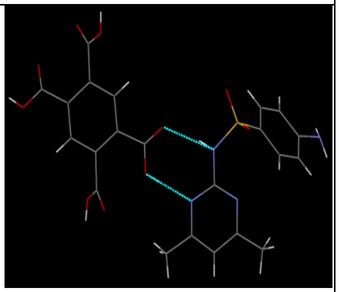<br>FEVKUO

2-amino-Py···COOH	2-amino-Py···COOH	2-amino-Py···COOH	2-amino-Py···COOH
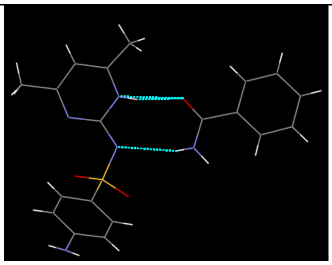<br>EXAPAV<br>2-amino-Py···CONH	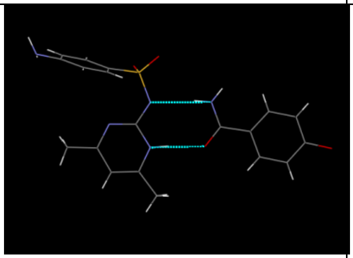<br>EXAPID<br>2-amino-Py···CONH	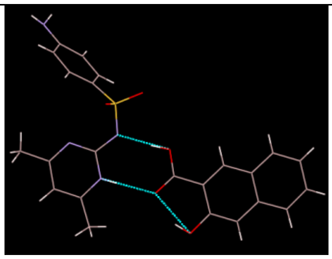<br>EXAPUP<br>2-amino-Py···COOH	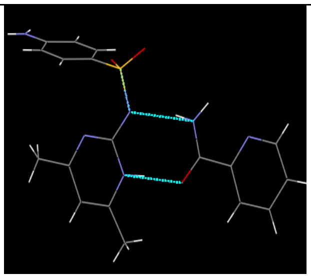<br>EXAPUZ<br>2-amino-Py···COOH
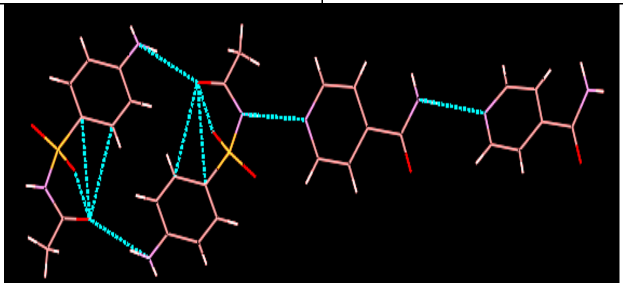<br>HOCCOT<br>N–H···N		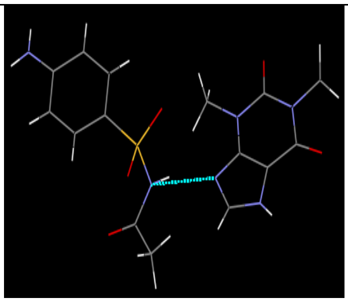<br>HOCCUZ<br>N–H···N	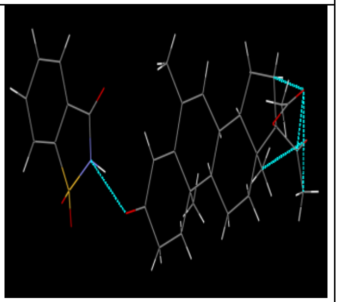<br>HORNEI<br>N–H···O
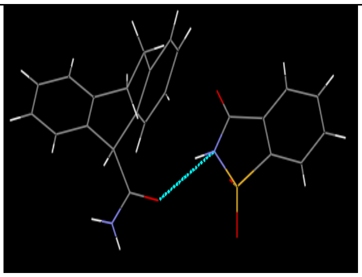<br>OTESAI<br>N–H···O	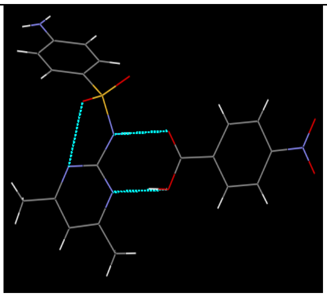<br>PAVKUU<br>2-amino-Py···COOH	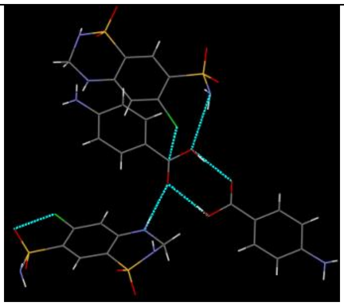<br>PIRLUZ<br>N–H···O	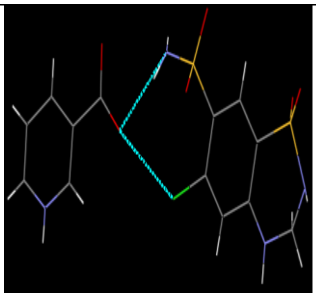<br>PIRXOF<br>N–H···O
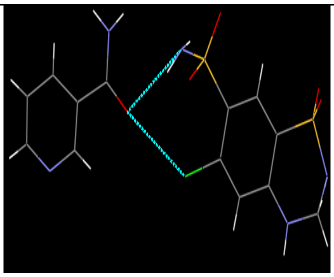<br>PIRQUL<br>2-amino-Py···COOH	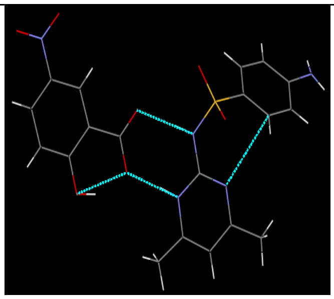<br>RILQUA<br>2-amino-Py···COOH	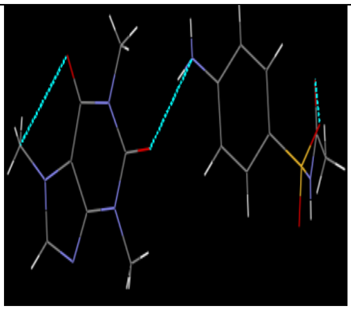<br>SACCAF<br>N–H···O	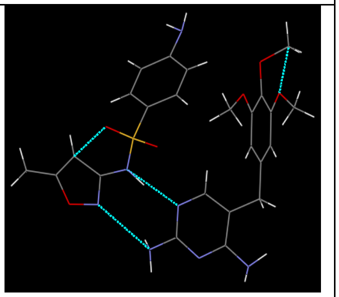<br>SMZPMP<br>2-amino-Py···hetrocylic N

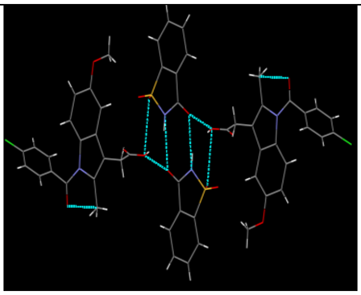 UFERED	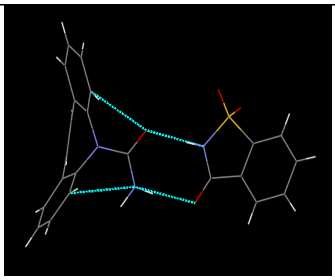 UNEZAO01<br>Amide dimer type	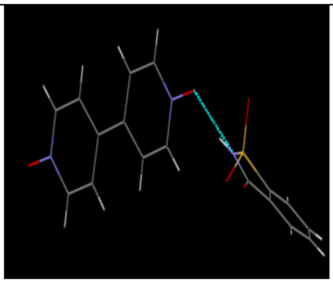 VIGFOH<br>SO_2NH_2···N-oxide	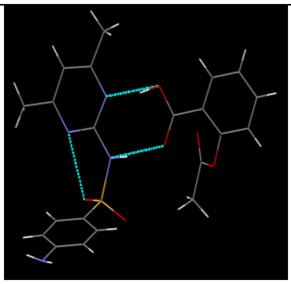 VUGMIT<br>2-amino-Py···COOH
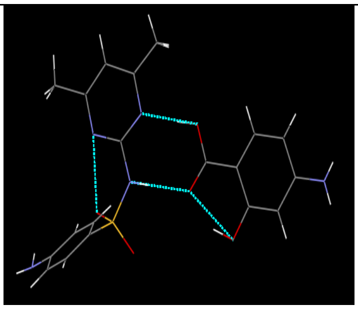 VUGMOJ<br>2-amino-Py···COOH	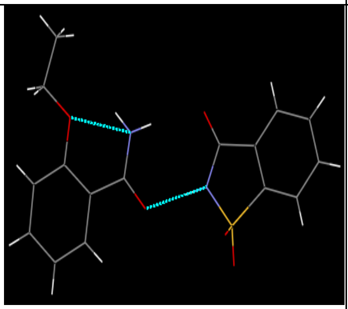 VUHFIO<br>N–H···O	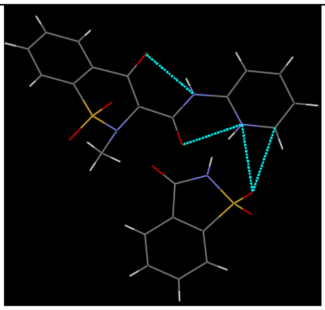 YANNEH<br>N–H···O	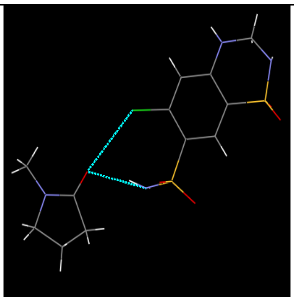 YESKIR<br>N–H···O
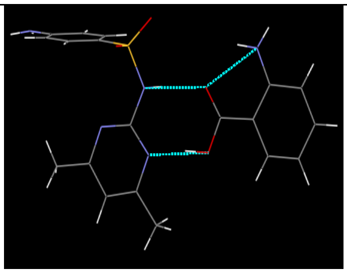 SORWEB<br>2-amino-Py···COOH	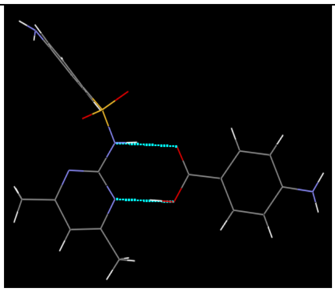 SORWIF<br>2-amino-Py···COOH	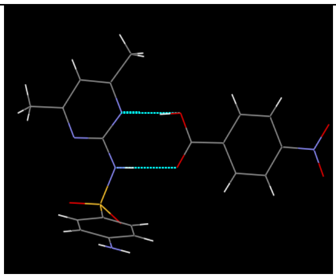 PAVKUU<br>2-amino-Py···COOH	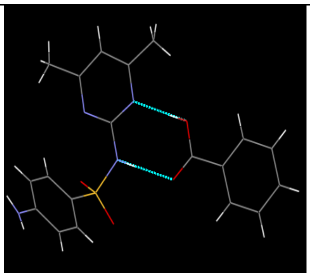 SABDOQ<br>2-amino-Py···COOH
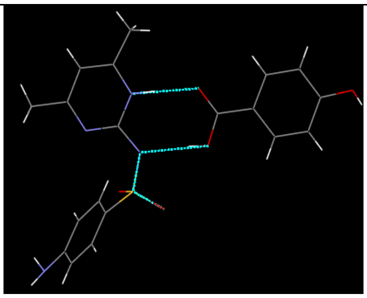 WXAMUM<br>2-amino-Py···COOH	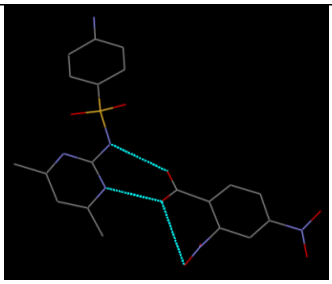 XEXCIE<br>2-amino-Py···COOH	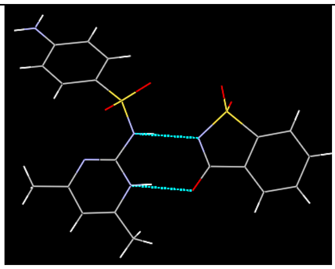 XOBCOH<br>2-amino-Py···COOH	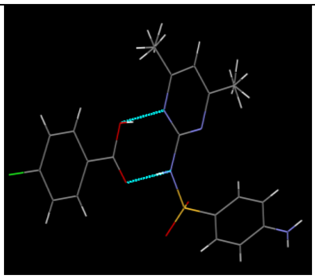 YIZQAA<br>2-amino-Py···CONH

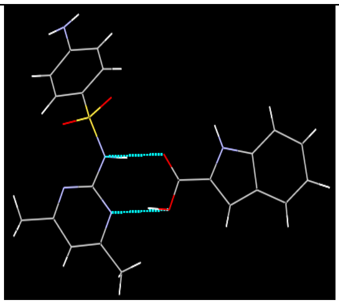<br>XEXCAE<br>2-amino-Py···COOH	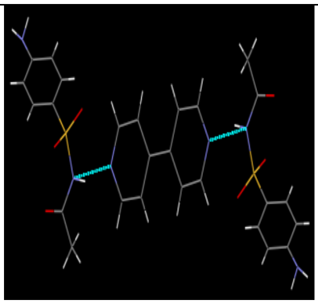<br>HOCCEJ<br>N–H···N	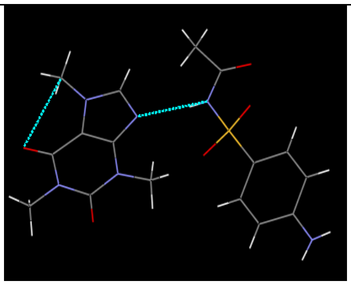<br>SACCAF01<br>N–H···N	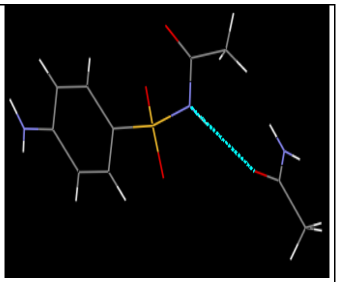<br>ZIMHIO01<br>N–H···O

(b)

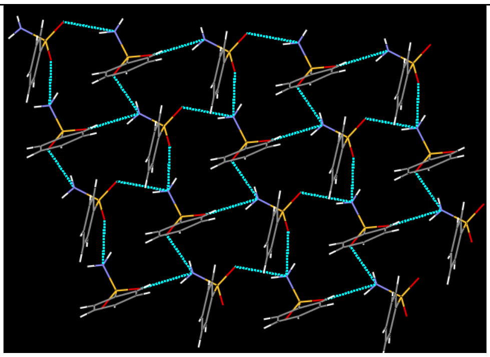<br>BSA catemer chains forms rings	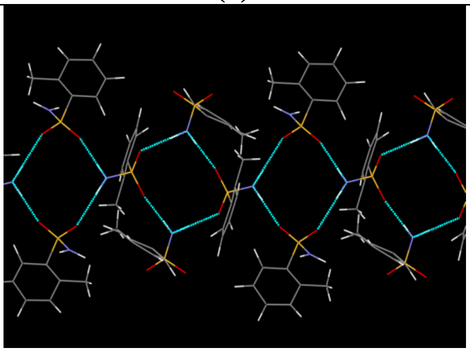<br>OTSA results ring motifs	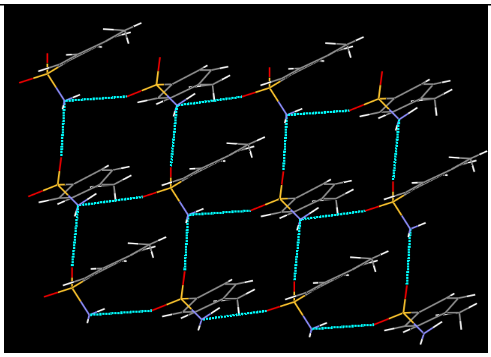<br>PTSA catemer chains forms rings
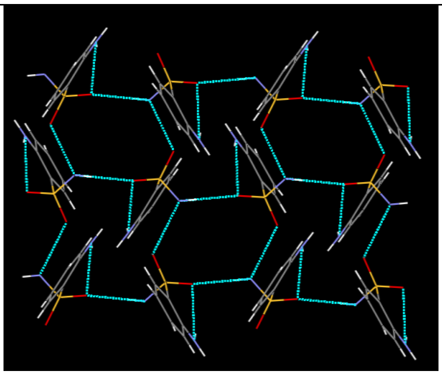<br>2ABSA catemer chains forms rings	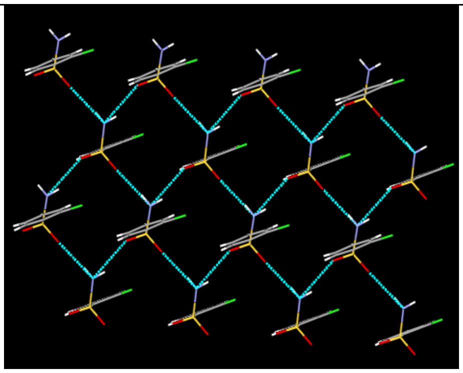<br>2ClBSA catemer chains forms rings	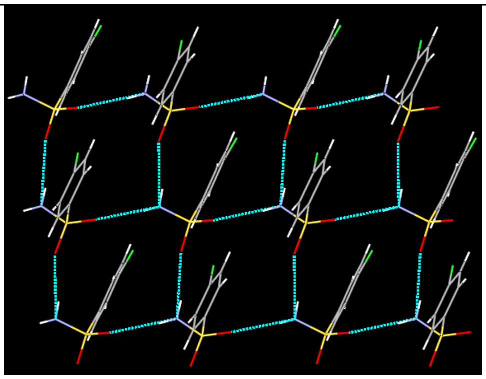<br>4ClBSA catemer chains forms rings

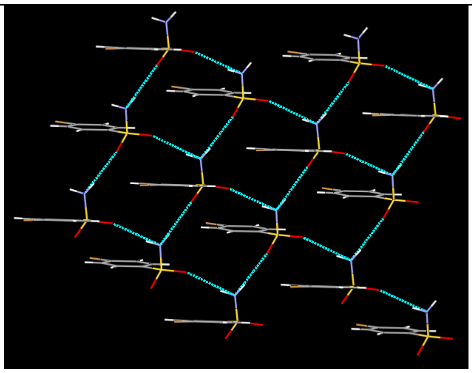<br>4BRBSA catemer chains forms rings	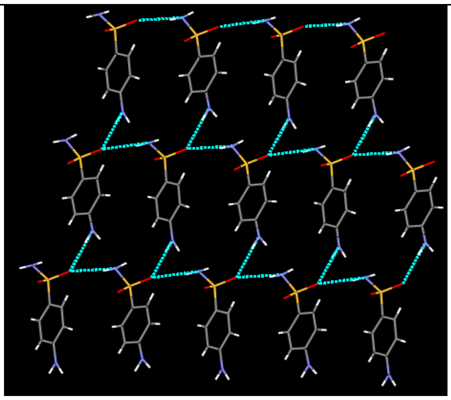<br>SNA-form I catemer chains forms rings	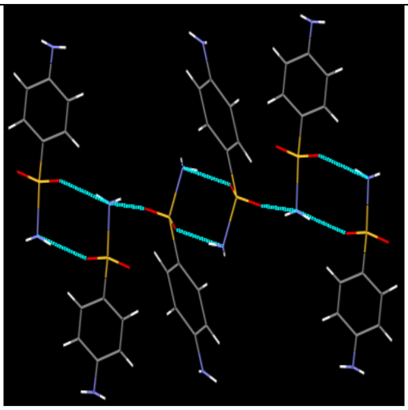<br>SNA form II dimer and discrete H bond
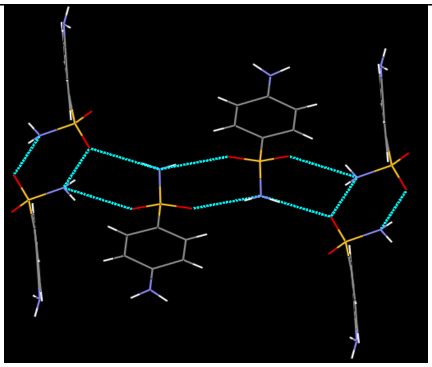<br>SNA Form III dimer followed by dimer	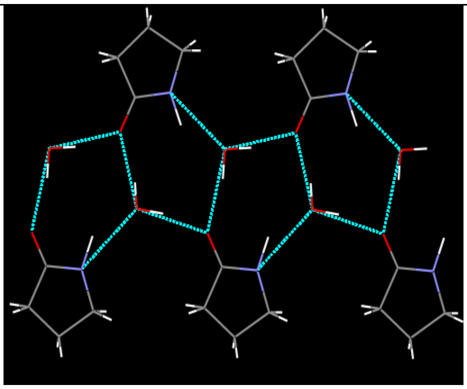<br>VLM lactam hydrate	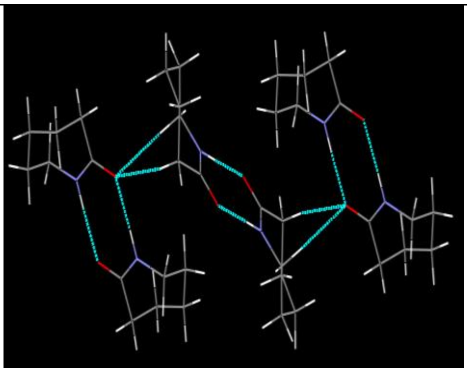<br>CPR dimer synthon
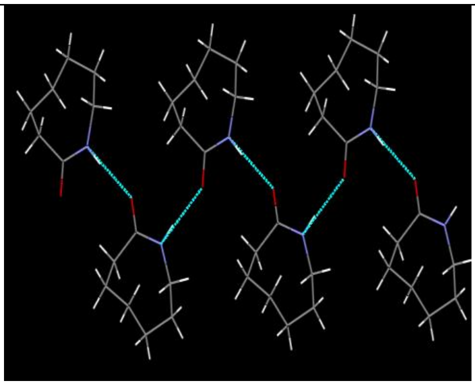<br>AZL shows catemer synthon		

(c)

Figure S5 (a) Reported primary sulfonamides cocrystal along with their Refcodes and interactions presented with coformers. (b) Secondary sulfonamides cocrystals along with their Refcodes and interactions presented with coformers. (c) All the sulfonamides as well as coformers crystals structures along with their

synthons reported in CSD database. The trend is that most of the sulfonamide structuress are contains catemer chains.

Table S3 The percentage of intermolecular interactions present in cocrystal structures from Hirshfeld surface analysis.

Cocrystal	N…H %	O…H %	X…H %	C…H %	Others %
2ABSA–CPR	4.3	29.3	-----	12.9	53.5
2ClBSA–VLM	2.8	25.6	11.7	5.7	54.2
2ClBSA–CPR	2.9	31.8	11.0	8.8	45.5
4BrBSA–VLM	4.3	29.9	13.5	2.5	49.8
4BrBSA–CPR	2.6	27.1	12.7	12.4	45.2
4ClBSA–VLM	3.4	29.4	12.4	6.4	48.4
4ClBSA–CPR	2.1	27.1	12.3	12.5	46.0
BSA–VLM	3.5	32.4	---	12.7	51.4
BSA–CPR	3.6	34.5	-----	10.1	51.8
BSA–AZL	2.3	32.7	----	12.3	52.7
OTSA–VLM	4.2	27.6	----	8.8	59.4
PTSA–VLM	4.4	31.0	----	7.6	57.0
SNA–CPR	5.2	29.9		11.2	53.7

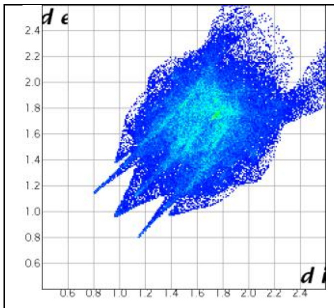	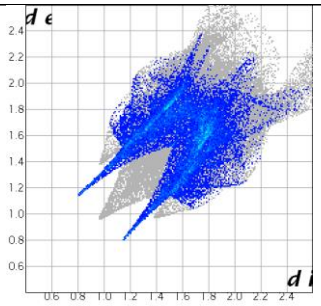	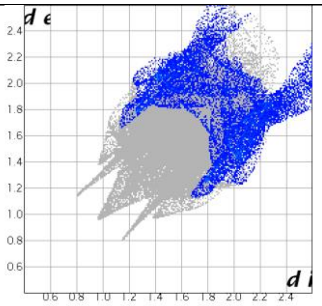	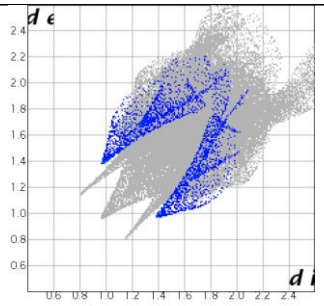
BSA–VLM All	BSA–VLM O···H	BSA–VLM C···H	BSA–VLM N···H
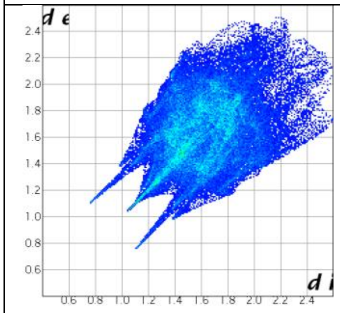	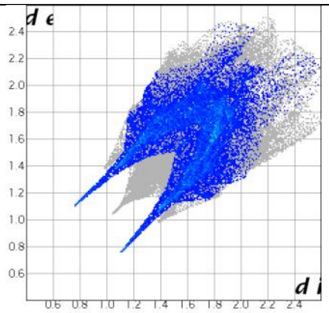	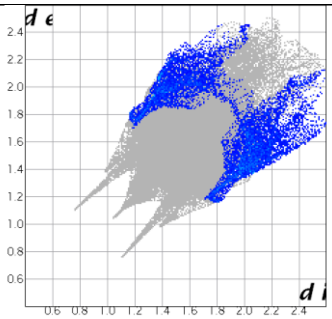	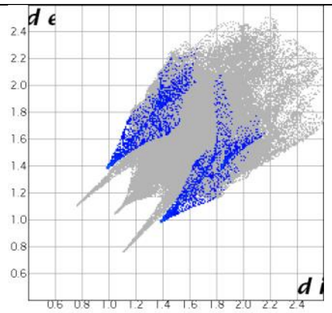
BSA–CPR All	BSA–CPR O···H	BSA–CPR C···H	BSA–CPR N···H
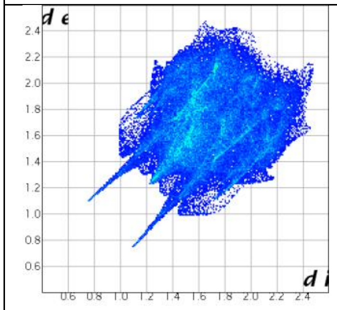	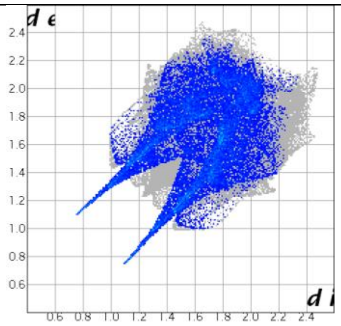	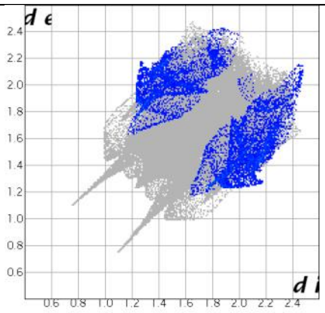	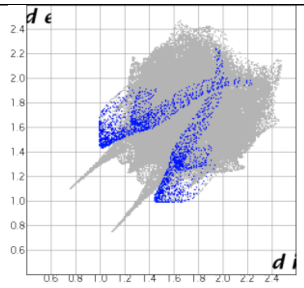
4ClBSA–CPR All	4ClBSA–CPR O···H	4ClBSA–CPR C···H	4ClBSA–CPR N···H
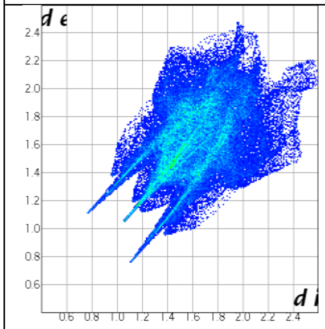	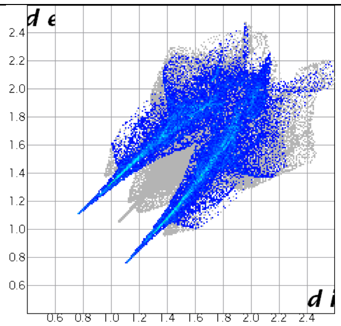	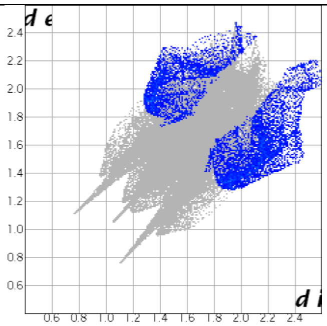	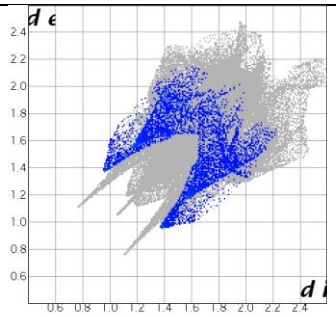
SNA–CPR All	SNA–CPR O···H	SNA–CPR C···H	SNA–CPR N···H

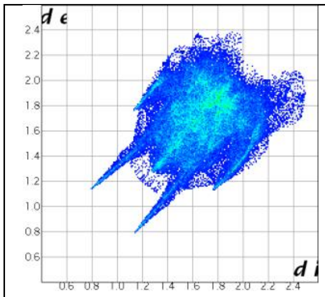<br>2ClBSA–VLM All	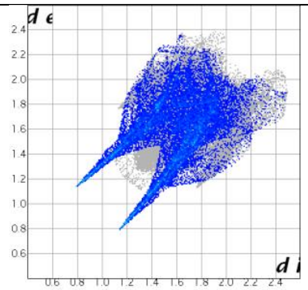<br>2ClBSA– VLM O···H	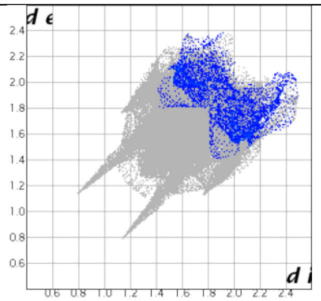<br>2ClBSA– VLM C···H	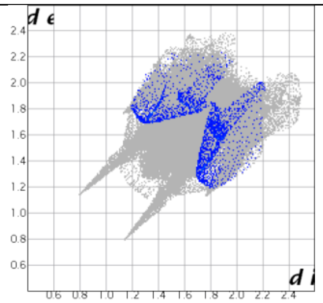<br>2ClBSA– VLM N···H
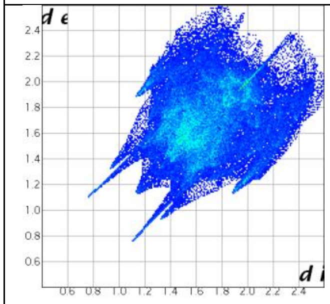<br>2BrBSA–VLM All	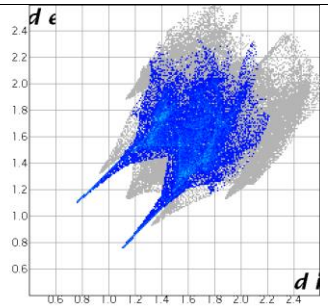<br>2BrBSA–VLM O···H	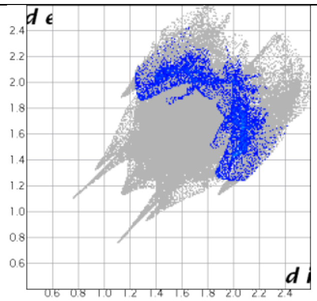<br>2BrBSA–VLM C···H	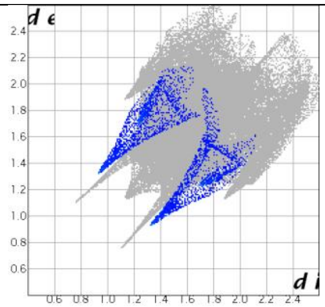<br>2BrBSA–VLM N···H
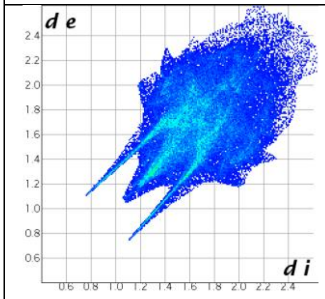<br>2ABSA–CPR All	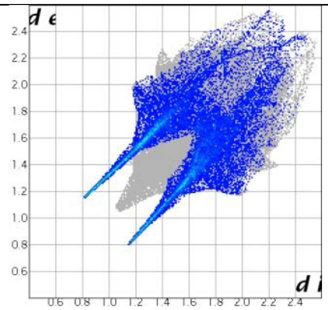<br>2ABSA–CPR O···H	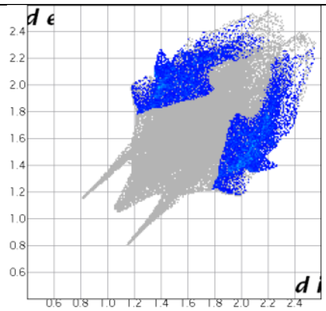<br>2ABSA–CPR C···H	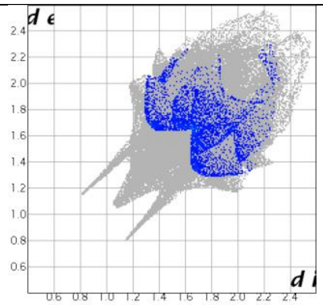<br>2ABSA–CPR N···H
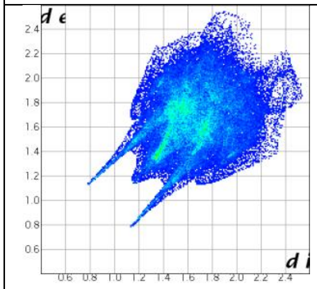<br>BSA–AZL All	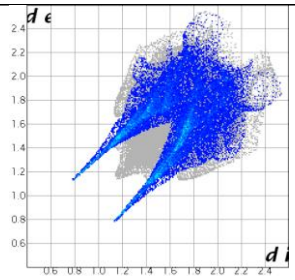<br>BSA–AZL O···H	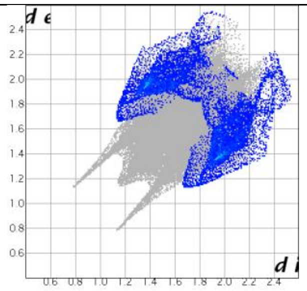<br>BSA–AZL C···H	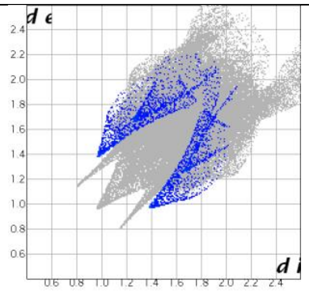<br>BSA–AZL N···H

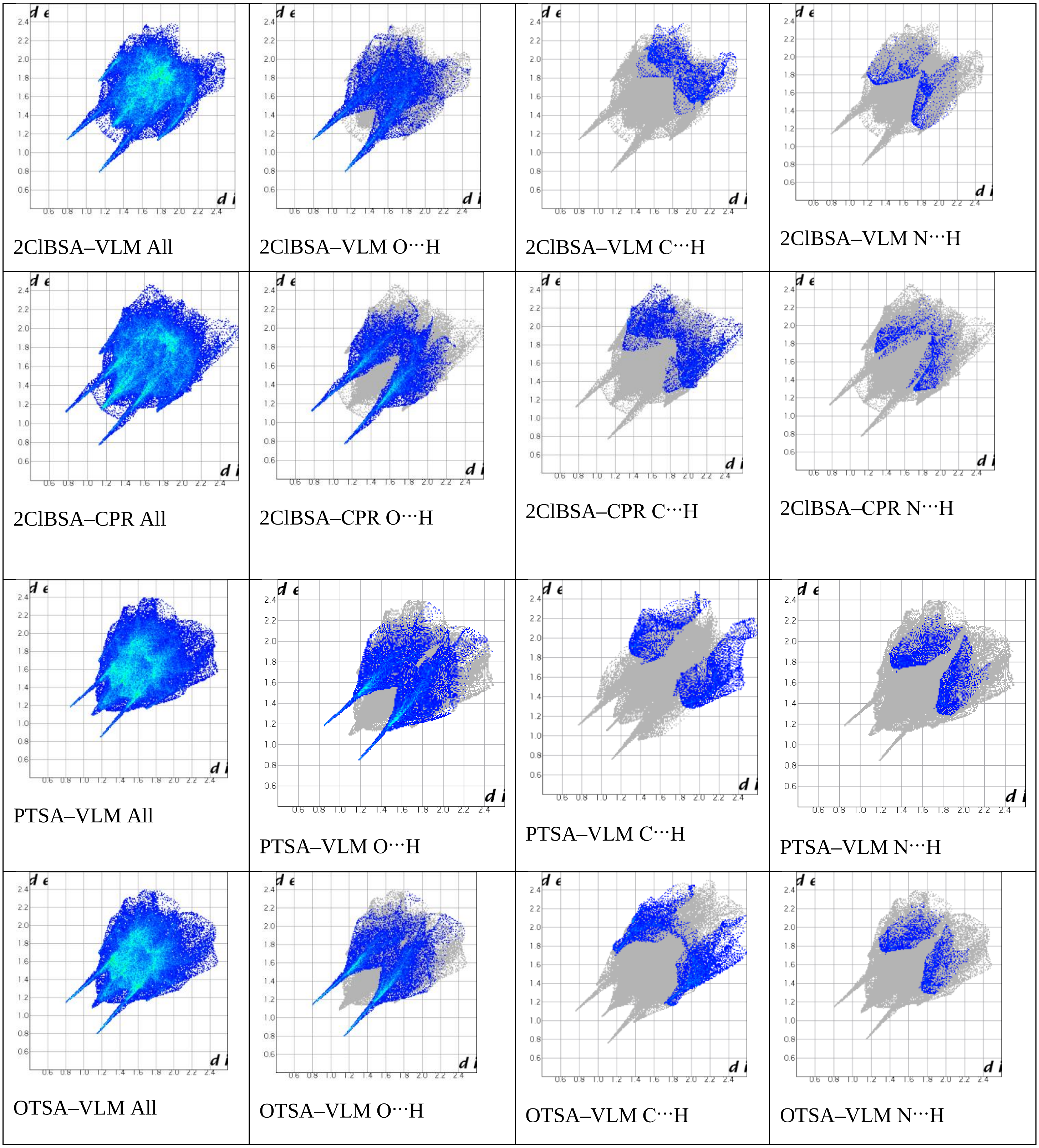

Figure S6 Hirshfeld 2D finger print plots of the interactions present in sulfonamide-lactam cocrystals.

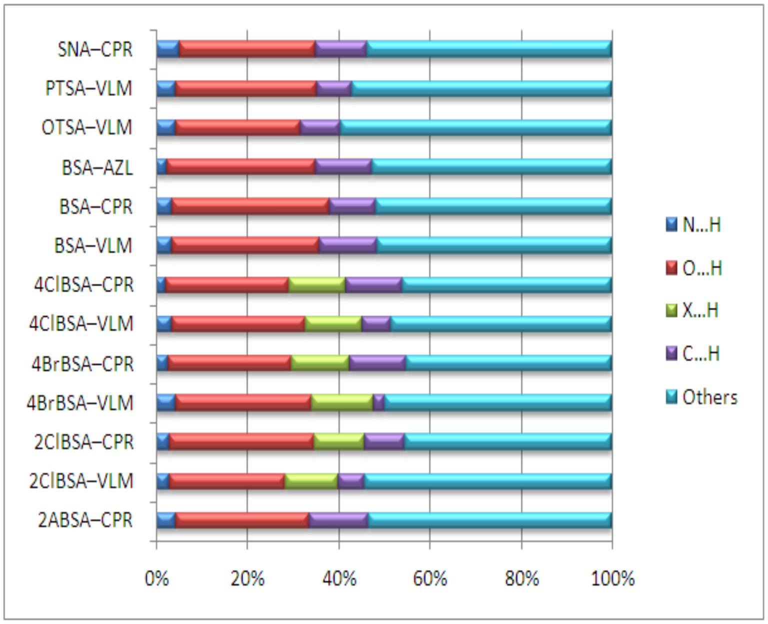

Figure S7 Percentage intermolecular contacts contributions to the Hirshfeld surface area in sulfonamide-lactam cocrystals.

Analysis of sulfonamide cocrystals by Vibrational Spectroscopy FT-IR spectroscopy is one of the prominent analytical methods for studying polymorphs/cocrystals in solid state chemistry. Hypsochromic /bathochromic shift of the vibrational frequency values for the ground adducts with starting individuals confirm new product formation. Broad N–H stretching vibrations for sulfonamides and lactams are observed in the range 3490–3330 cm^{-1} respectively due to intermolecular hydrogen bonding. Asymmetric and symmetric S=O stretching vibrations appear as strong absorption peaks in the range 1344–1305 cm^{-1} and 1178–1144 cm^{-1}, see Table S4. All the spectra comparisons for sulfonamide cocrystals were kept in Figure S7, SI.

Table S4 IR frequency (cm^{-1}) of sulfonamide-lactam cocrystals

Single/binary components	S=O asym/sym stretch (cm^{-1})	N–H stretch (cm^{-1})	N–H bend (cm^{-1})	C=O stretch (cm^{-1})
BSA	1334.1/1159.5	3355.2	1480.4	-----
BSA–VLM	1334.2/1155.9	3352.5	1499.0	1634.7
BSA–CPR	1332.9/1160.6	3352.1	1481.7	1649.0
BSA–AZL	1344.1/1161.5	3292.3	1447.5	1630.3
2ClBSA	1333.2/1177.5	3355.1	1453.2	-----
2ClBSA–VLM	1338.0/1159.7	3357.2	1453.2	1651.4

2ClBSA–CPR	1326.4/1157.5	3348.3	1487.5	1644.7
4BrBSA	1328.0/1148.2	3331.1	1469.9	-----
4BrBSA–VLM	1349.2/1165.8	3331.0	1468.7	1621.0
4BrBSA–CPR	1335.5/1160.0	3370.2	1488.1	1628.6
4ClBSA	1331.7/1151.3	3334.5	1475.0	-----
4ClBSA–VLM	1348.4/1162.7	3328.8	1468.5	1622.8
4ClBSA–CPR	1341.1/1178.7	3367.5	1476.7	1631.3
2ABSA	1308.3/1144.4	3481.3	1479.9	-----
2ABSA–CPR	1312.2/1140.2	3452.9	1480.9	1644.0
OTSA	1316.3/1148.7	3390.5	1472.3	-----
OTSA–VLM	1337.0/1161.0	3380.2	1476.1	1663.9
PTSA	1323.9/1150.3	-----	1495.7	-----
PTSA–VLM	1340.2/1156.5	3296.3	1500.3	1659.3
SNA	1313.8/1146.1	3478.1	1504.0	-----
SNA–CPR	1309.5/1145.6	3465.7	-----	1636.0
VLM	-----	3273.2	1497.5	1632.4
CPR	-----	3429.9	1487.2	1636.0
AZL	-----	-----	1475.7	1659.9

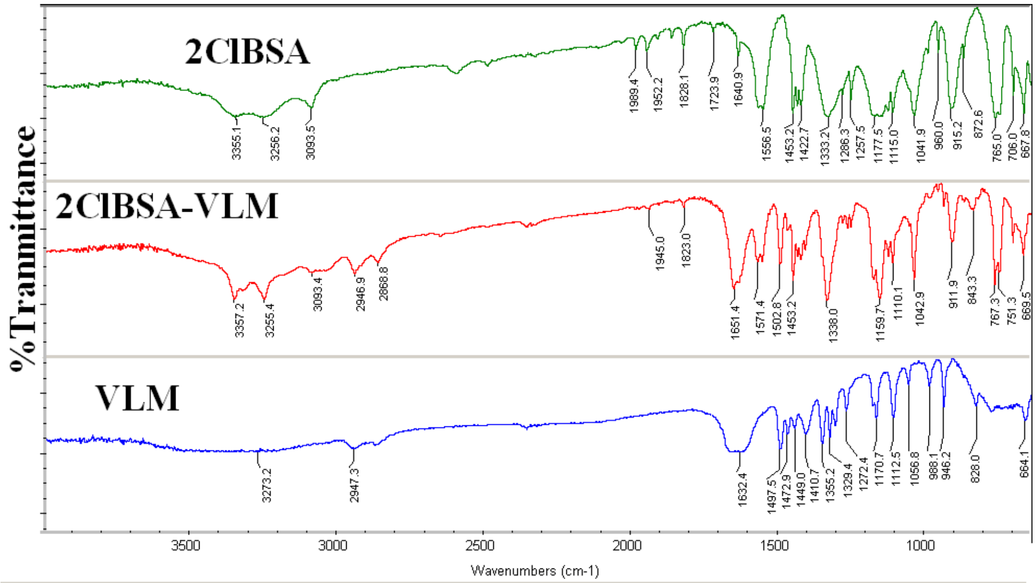
2ClBSA
2ClBSA-VLM
VLM
% Tranmittance
3500
3000
2500
2000
1500
1000
Wavenumbers (cm-1)

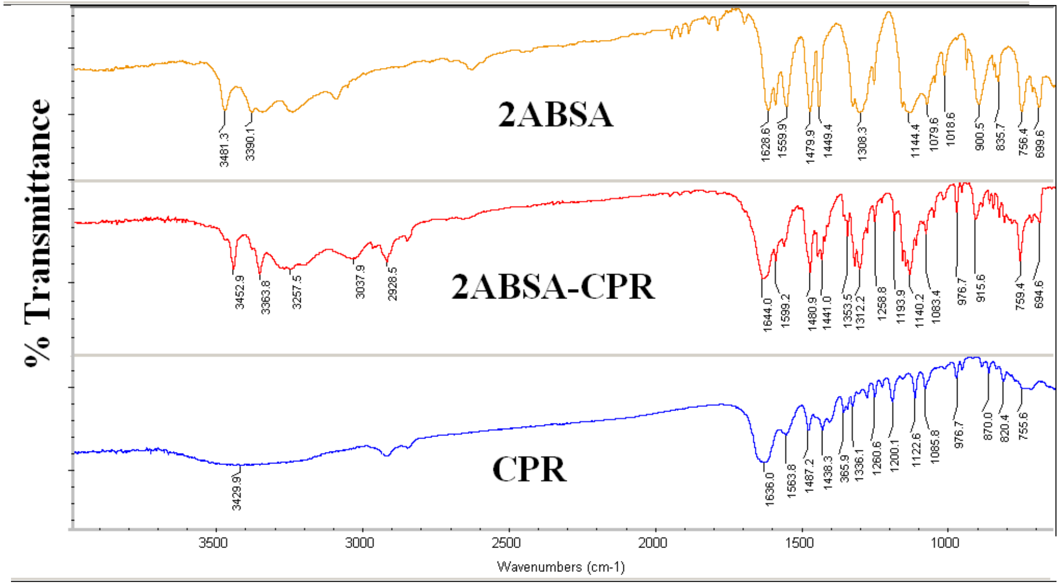
2ABSA
2ABSA-CPR
CPR
% Transmittance
3500
3000
2500
2000
1500
1000
Wavenumbers (cm-1)

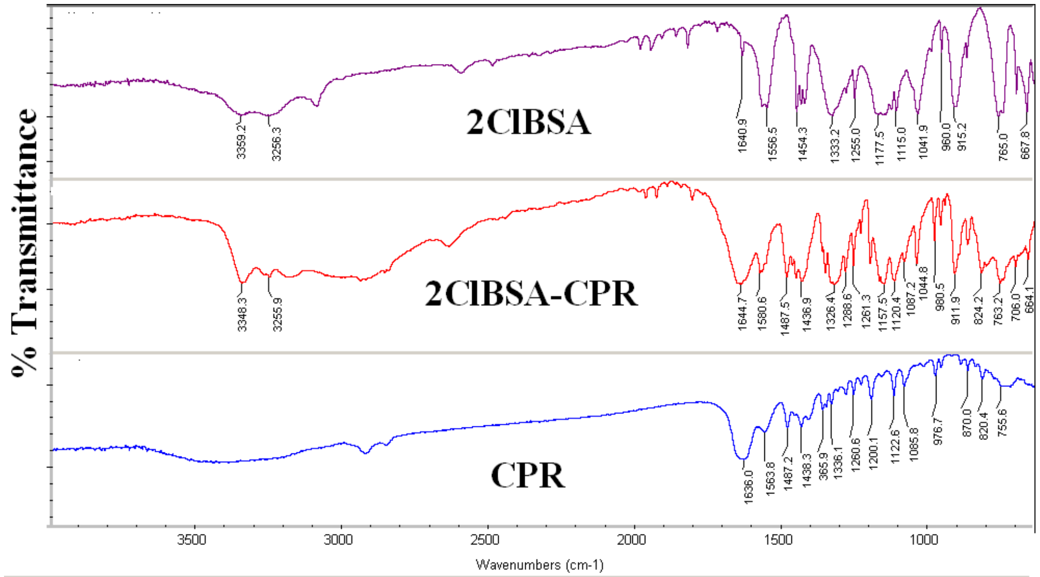
% Transmittance
2ClBSA
2ClBSA-CPR
CPR
3500
3000
2500
2000
1500
1000
Wavenumbers (cm-1)

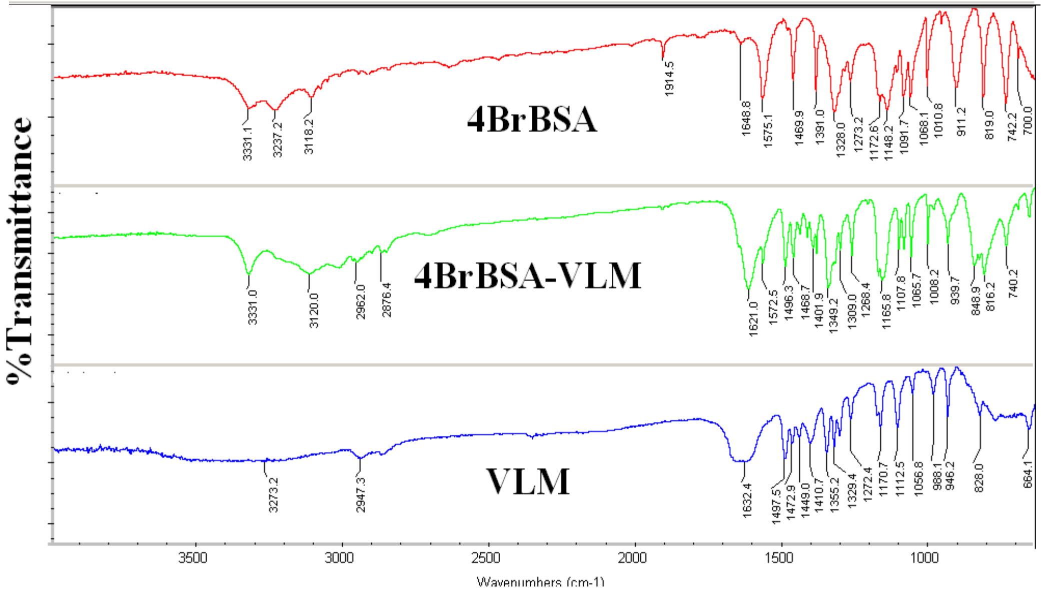
%Transmittance
4BrBSA
4BrBSA-VLM
VLM
3500
3000
2500
2000
1500
1000
Wavenumbers (cm-1)

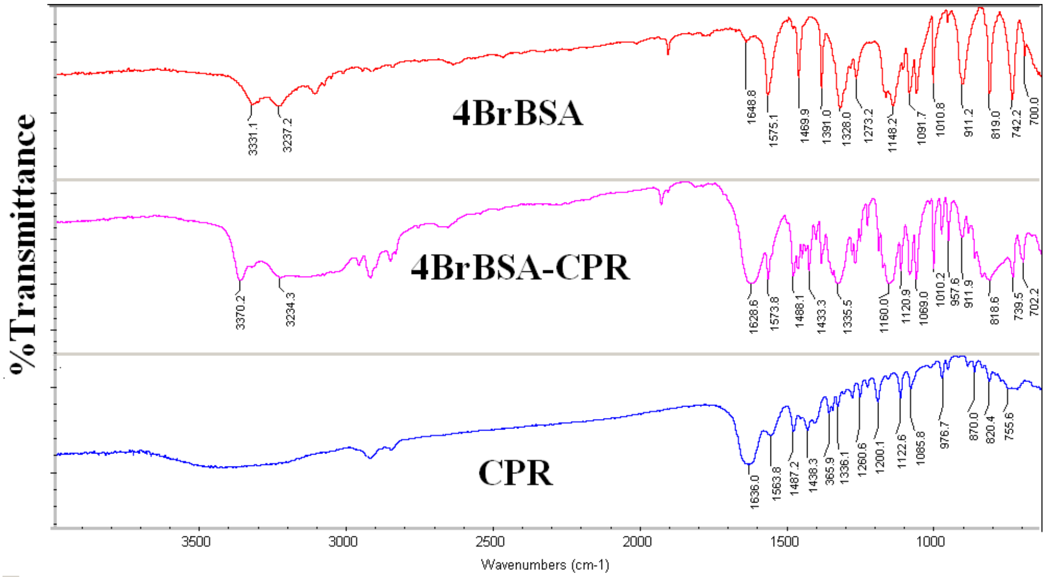
% Transmittance
4BrBSA
3331.1
3237.2
1648.8
1575.1
1469.9
1391.0
1328.0
1273.2
1148.2
1091.7
1010.8
911.2
819.0
742.2
700.0
4BrBSA-CPR
3370.2
3234.3
1626.6
1573.8
1488.1
1433.3
1335.5
1160.0
1120.9
1069.0
1010.2
957.6
911.9
818.6
739.5
702.2
CPR
1636.0
1563.8
1487.2
1438.3
365.9
1336.1
1260.6
1200.1
1122.6
1085.8
976.7
870.0
820.4
755.6
3500
3000
2500
2000
1500
1000
Wavenumbers (cm-1)

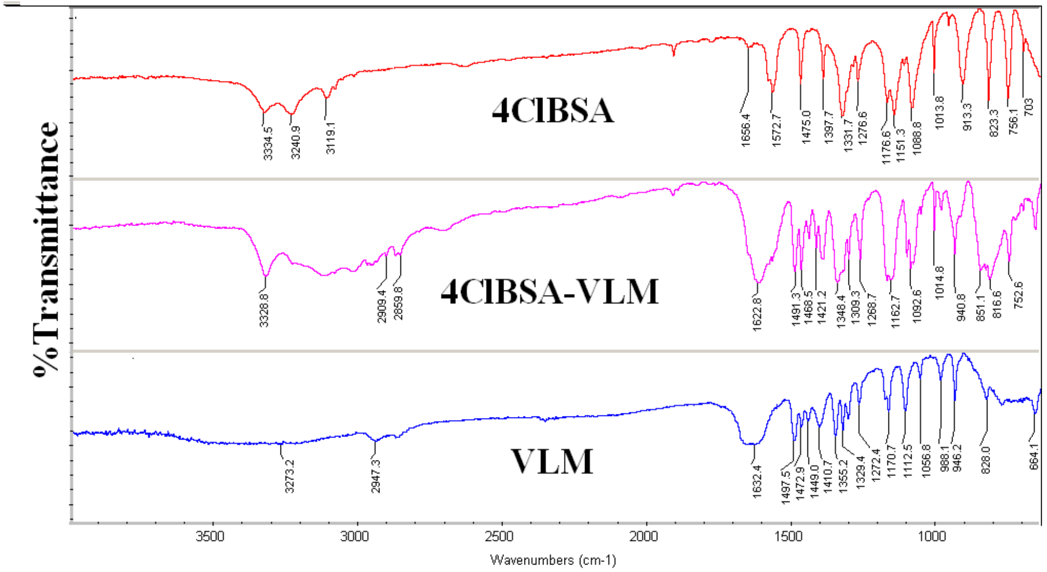
% Transmittance
4ClBSA
3334.5
3240.9
3119.1
1656.4
1572.7
1475.0
1397.7
1331.7
1276.6
1176.6
1151.3
1088.8
1013.8
913.3
823.3
756.1
703
4ClBSA-VLM
3328.8
2909.4
2859.8
1622.8
1491.3
1468.5
1421.2
1348.4
1309.3
1268.7
1162.7
1092.6
1014.8
940.8
851.1
816.6
752.6
VLM
3273.2
2947.3
1632.4
1497.5
1472.9
1449.0
1410.7
1355.2
1329.4
1272.4
1170.7
1112.5
1056.8
988.1
946.2
828.0
664.1
3500
3000
2500
2000
1500
1000
Wavenumbers (cm-1)

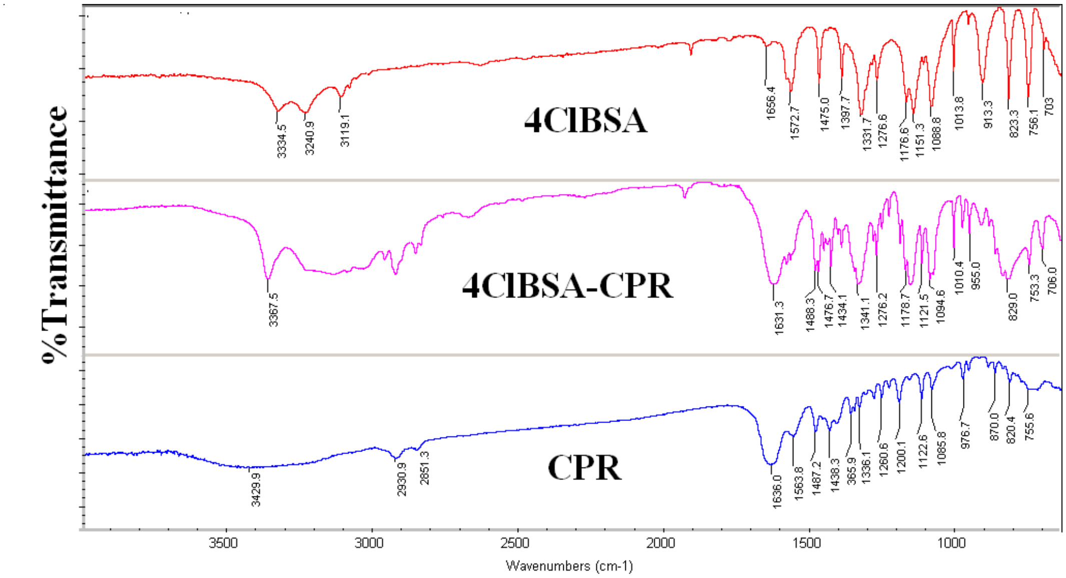
% Transmittance
4ClBSA
3334.5
3240.9
3119.1
1656.4
1572.7
1475.0
1397.7
1331.7
1276.6
1176.6
1151.3
1088.8
1013.8
913.3
823.3
756.1
703
4ClBSA-CPR
3367.5
1631.3
1488.3
1476.7
1434.1
1341.1
1276.2
1178.7
1121.5
1094.6
1010.4
955.0
829.0
753.3
706.0
CPR
3429.9
2930.9
2851.3
1636.0
1563.8
1487.2
1438.3
365.9
1336.1
1260.6
1200.1
1122.6
1085.8
976.7
870.0
820.4
755.6
3500
3000
2500
2000
1500
1000
Wavenumbers (cm-1)

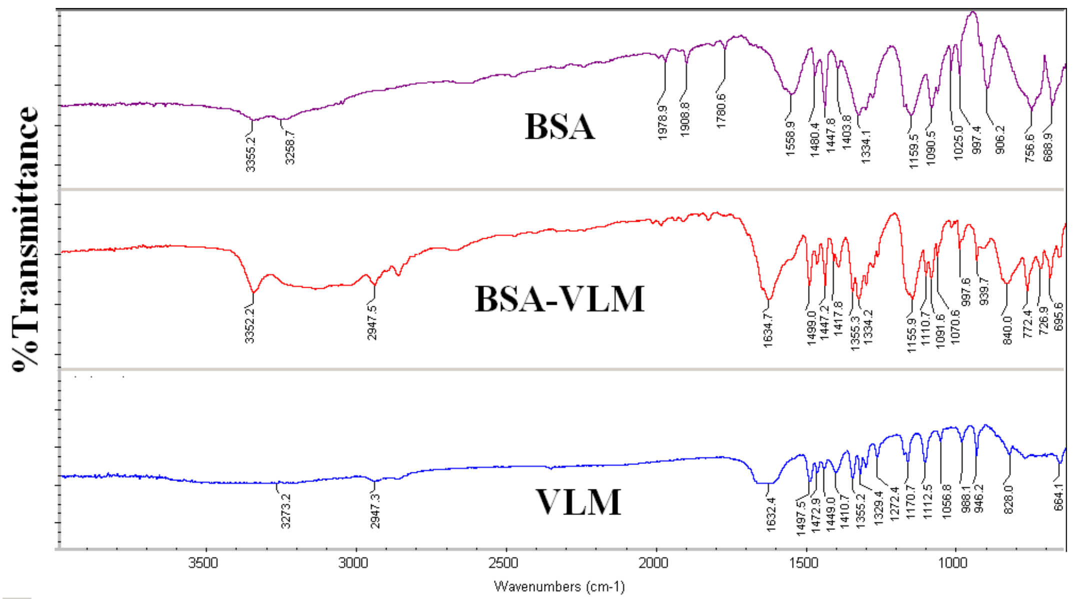
%Transmittance
BSA
BSA-VLM
VLM
Wavenumbers (cm-1)

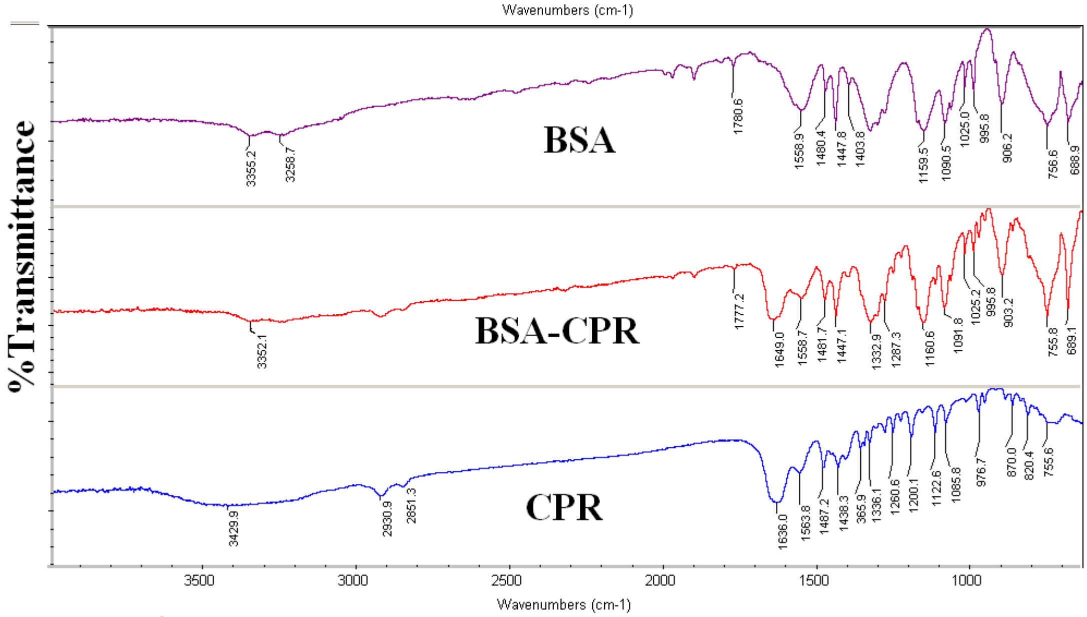
%Transmittance
BSA
BSA-CPR
CPR
Wavenumbers (cm-1)

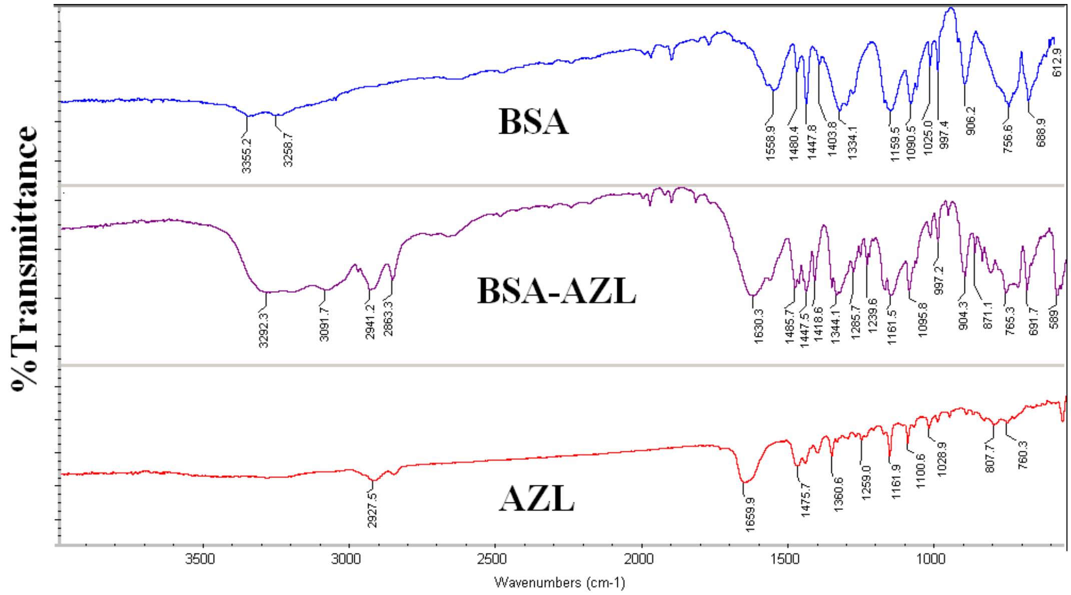
%Transmittance
BSA
3355.2
3258.7
1558.9
1480.4
1447.8
1403.8
1334.1
1159.5
1090.5
1025.0
997.4
906.2
756.6
688.9
612.9
BSA-AZL
3292.3
3091.7
2941.2
2863.3
1630.3
1485.7
1447.5
1418.6
1344.1
1285.7
1239.6
1161.5
1095.8
997.2
904.3
871.1
765.3
691.7
589
AZL
2927.5
1659.9
1475.7
1360.6
1259.0
1161.9
1100.6
1028.9
807.7
760.3
3500
3000
2500
2000
1500
1000
Wavenumbers (cm-1)

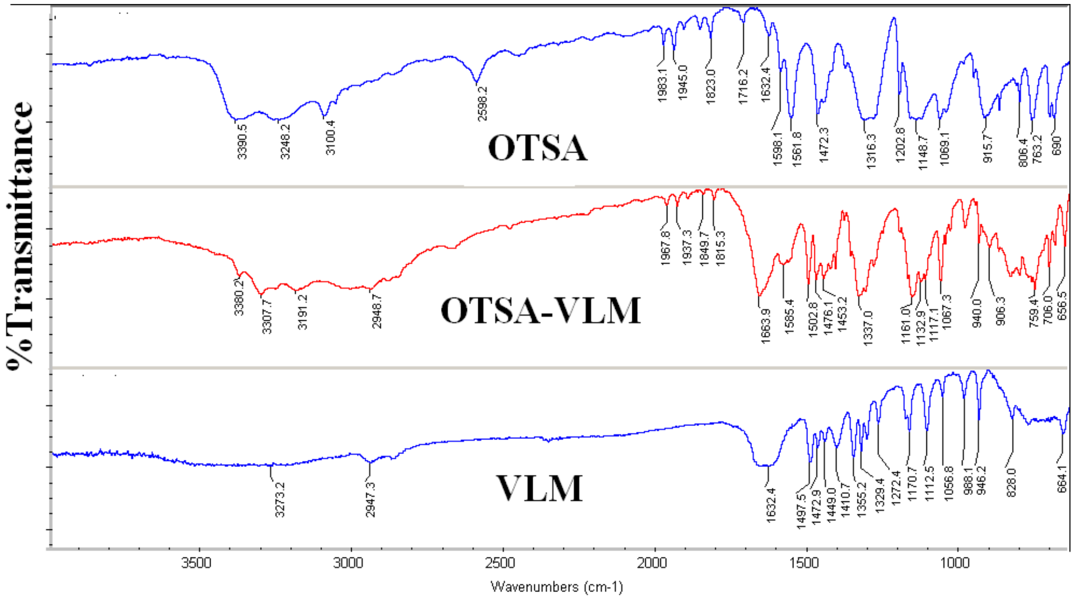
%Transmittance
OTSA
3390.5
3248.2
3100.4
2598.2
1983.1
1945.0
1823.0
1716.2
1632.4
1598.1
1561.8
1472.3
1316.3
1202.8
1148.7
1069.1
915.7
806.4
763.2
690
OTSA-VLM
3380.2
3307.7
3191.2
2948.7
1967.8
1937.3
1849.7
1815.3
1663.9
1585.4
1502.8
1476.1
1453.2
1337.0
1161.0
1132.9
1117.1
1067.3
940.0
906.3
759.4
706.0
656.5
VLM
3273.2
2947.3
1632.4
1497.5
1472.9
1449.0
1410.7
1355.2
1329.4
1272.4
1170.7
1112.5
1056.8
988.1
946.2
828.0
664.1
3500
3000
2500
2000
1500
1000
Wavenumbers (cm-1)

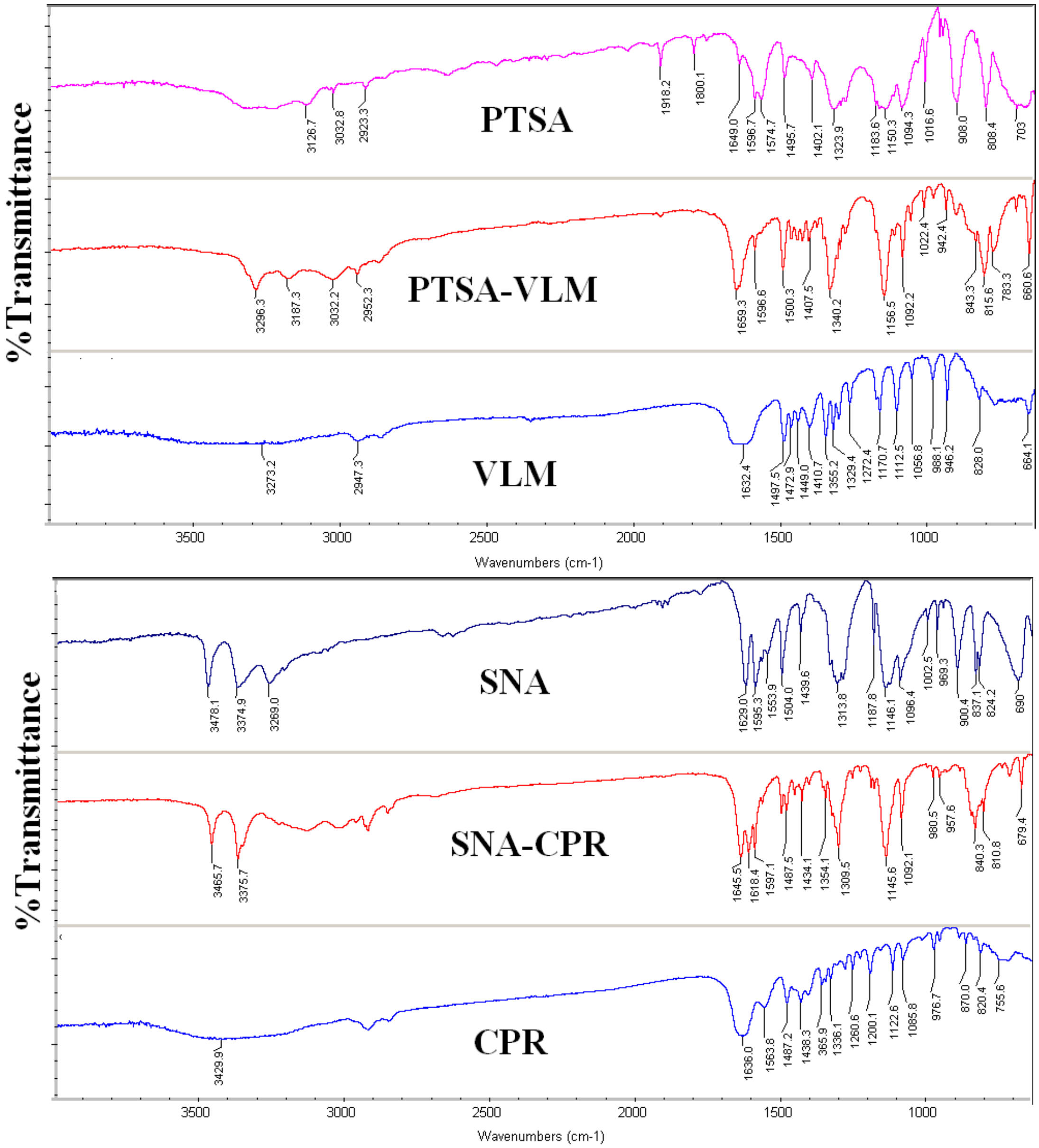

Figure S8 IR spectra of all sulfopnamide–lactam cocrystals along with starting materials.

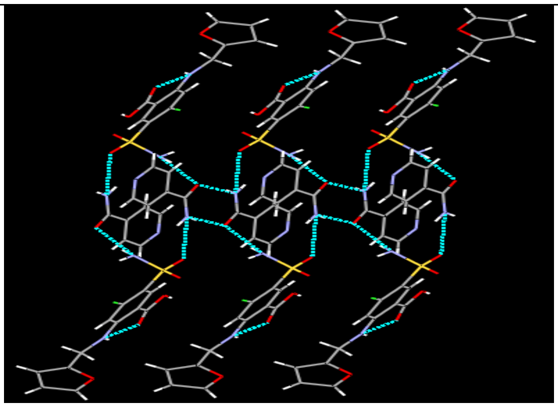

Furosemide-Isonicotinamide (1:1)

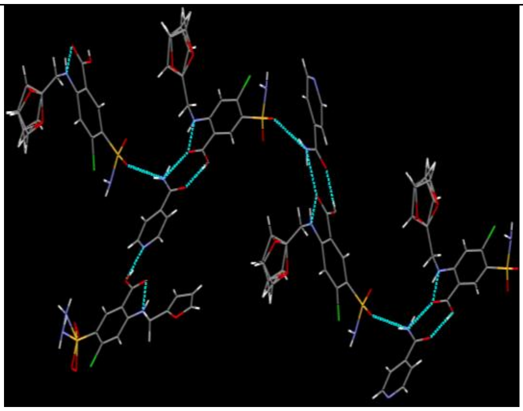

Furosemide-Isonicotinamide (2:1)

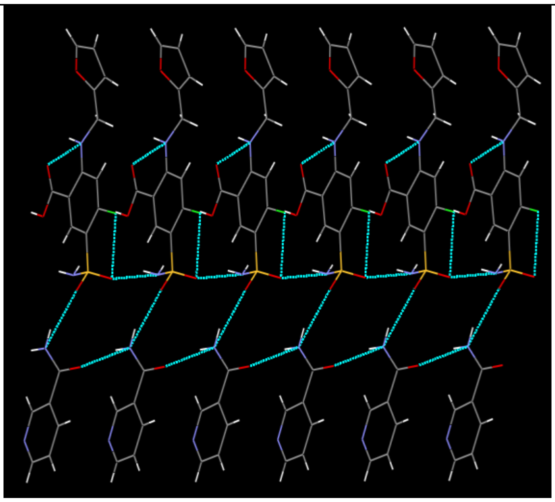

Furosemide-Nicotinamide (IV)

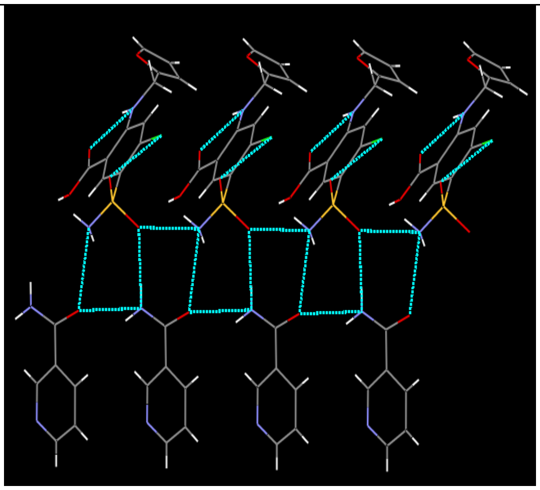

Furosemide-Nicotinamide (III)

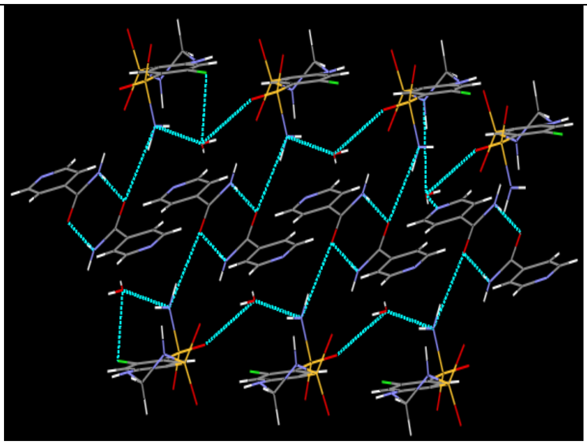

Hydrochlorothiazide-Isonicotinamide

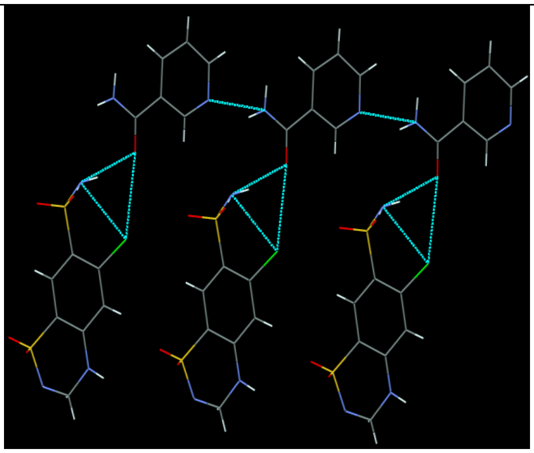

Hydrochlorothiazide-Nicotinamide

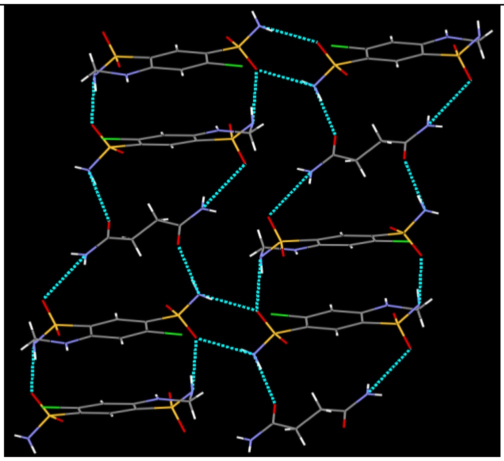<br>Hydrochlorothiazide-succinamide	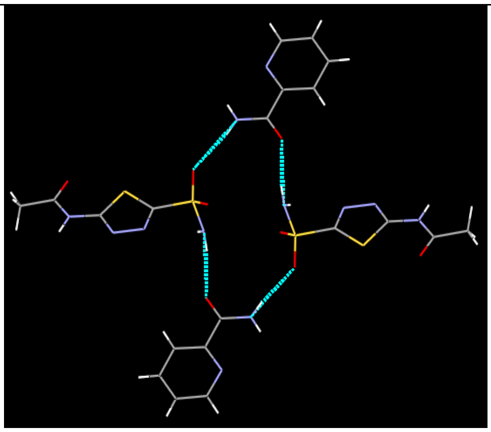<br>Acetazolamide-Picolinamide
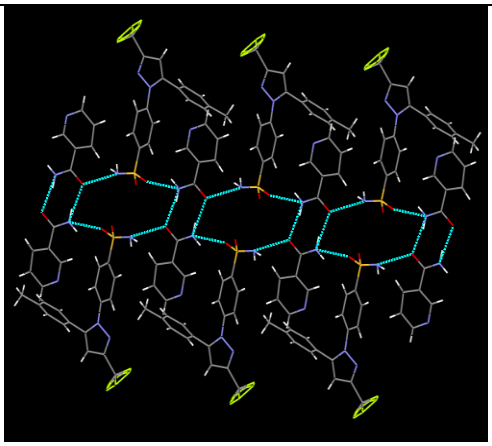<br>Celecoxib-Nicotinamide	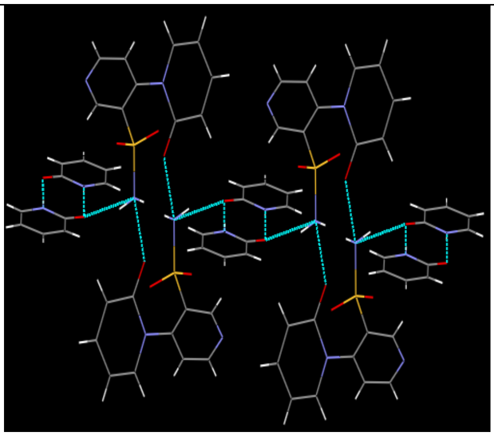<br>Sulfonamide-2Pyridone

Figure S9 2D Packing visualization of the primary sulfonamides with amide coformers reported with sulfonamide-carboxamide heterosynthon in the CSD database.
